# The farnesyltransferase β‐subunit RAM1 regulates localization of RAS proteins and appressorium‐mediated infection in *Magnaporthe oryzae*

**DOI:** 10.1111/mpp.12838

**Published:** 2019-06-27

**Authors:** Ahmed Aboelfotoh Hendy, Junjie Xing, Xiaoyang Chen, Xiao‐Lin Chen

**Affiliations:** ^1^ The Provincial Key Lab of Plant Pathology of Hubei Province, College of Plant Science and Technology Huazhong Agricultural University Wuhan 430070 China; ^2^ State Key Laboratory of Hybrid Rice Hunan Hybrid Rice Research Center Changsha 410125 China; ^3^ Department of Agricultural Botany, Faculty of Agriculture (Saba Basha) Alexandria University Alexandria 21531 Egypt

**Keywords:** appressorium formation, cAMP signalling pathway, farnesylation, RAS protein, rice blast fungus

## Abstract

Post‐translational farnesylation can regulate subcellular localization and protein–protein interaction in eukaryotes. The function of farnesylation is not well identified in plant pathogenic fungi, particularly during the process of fungal infection. Here, through functional analyses of the farnesyltransferase β‐subunit gene, *RAM1*, we examine the importance of protein farnesylation in the rice blast fungus *Magnaporthe oryzae*. Targeted disruption of *RAM1* resulted in the reduction of hyphal growth and sporulation, and an increase in the sensitivity to various stresses. Importantly, loss of *RAM1* also led to the attenuation of virulence on the plant host, characterized by decreased appressorium formation and invasive growth. Interestingly, the defect in appressoria formation of the Δ*ram1* mutant can be recovered by adding exogenous cAMP and IBMX, suggesting that *RAM1* functions upstream of the cAMP signalling pathway. We found that two Ras GTPases, RAS1 and RAS2, can interact with Ram1, and their plasma membrane localization was regulated by Ram1 through their C‐terminal farnesylation sites. Adding a farnesyltransferase inhibitor Tipifarnib can result in similar defects as in Δ*ram1* mutant, including decreased appressorium formation and invasive growth, as well as mislocalized RAS proteins. Our findings indicate that protein farnesylation regulates the RAS protein‐mediated signaling pathways required for appressorium formation and host infection, and suggest that abolishing farnesyltransferase could be an effective strategy for disease control.

## Introduction

Protein farnesylation is one type of prenylation modification, which is required for the proper localization of many proteins in signal transduction, including Ras proteins (Maurer‐Stroh *et al.*, 2003; Zhang and Casey, 1996). Farnesylation modifies membrane‐associated proteins through their C‐terminal Caa X‐motif (C is cysteine, A is an aliphatic residue, X can be variable amino acids) on the cysteine residue (Fu and Casey, 1999). During modification, the farnesylation site of the target protein can be linked by a 15‐carbon isoprenoid farnesyl moiety, which is catalysed by farnesyltransferase (FTase) (Casey and Seabra, 1996). The FTase is an αβ heterodimer composed of an essential α‐subunit Ram2 and a non‐essential β‐subunit Ram1 (Casey and Seabra, 1996; Maurer‐Stroh *et al.*, 2003).

Although deletion of the FTase α‐subunit is lethal in fungi such as *Saccharomyces cerevisiae* and *Candida albicans* (He *et al.*, 1991; Song and White, 2003), functions of farnesylation have been investigated by successful deletion of the FTase β‐subunit in several fungi, including *S. cerevisiae*, *C. albicans*, *Schizosaccharomyces pombe*, *Cryptococcus neoformans* and *Aspergillus fumigatus* (He *et al.*, 1991; Norton *et al.*, 2017; Vallim *et al.*, 2004; Yang *et al.*, 2000). In *S. cerevisiae*, the *ram1* null mutants were severely defective in growth at low temperatures and cannot grow at 37 °C (He *et al.*, 1991). In *S. pombe*, deletion of *cpp1+*, a homologue of *RAM1*, resulted in rounded or irregular cell morphology (Yang *et al.*, 2000). *RAM1* is also found to be required for virulence in *C. neoformans* and *A. fumigatus* (Norton *et al.*, 2017; Vallim *et al.*, 2004). In plant pathogenic fungi *Ustilago maydis* and *Ustilago hordei*, the a‐factor lipopeptide mating pheromones were found to be farnesylated, which is important for their functions (Caldwell *et al.*, 1995; Kosted *et al.*, 2000; Spellig *et al.*, 1994). However, the functions of farnesylation, especially during the infection process, are still largely unknown in plant pathogenic fungi.

*Magnaporthe oryzae* is a hemibiotrophic ascomycete fungus that destroys a massive amount of rice production and has become a model plant fungal pathogen (Wilson and Talbot, 2009). *Magnaporthe oryzae* produces an infection structure called the appressorium that enables it to penetrate host plant cells and initiate invasive growth (Yan and Talbot, 2016). Several signalling pathways, especially the cAMP‐dependent protein kinase A (cAMP/PKA), Pmk1 mitogen‐activated protein kinase (MAPK), and target of rapamycin (TOR) signalling pathways, are involved in appressorium morphogenesis and penetration (Marroquin‐Guzman and Wilson, 2015; Mitchell and Dean, 1995; Thines *et al.*, 2000; Xu and Hamer, 1996).

RAS (rat sarcoma) proteins are small GTP‐binding proteins controlling the switch of the active GTP and inactive GDP‐bound statuses, which are usually located on the plasma membrane and respond to external stimuli to activate various downstream signalling pathways for cellular responses (Milburn *et al.*, 1990). In *S. cerevisiae*, two RAS proteins, Ras1 and Ras2, both control the cAMP‐PKA signalling pathway (Tamanoi, 2011). RAS2 is also reported to function upstream of both of the cAMP and Pmk1‐MAPK signalling pathways in *M. oryzae* to regulate appressorium formation (Zhou *et al.*, 2014). Normally, the location of the Ras proteins on the plasma membrane is essential for their functions.

In this study, we attempted to determine the role of protein farnesylation in the development and infection of *M. oryzae*. We successfully deleted the *M. oryzae* farnesyltransferase β‐subunit gene *RAM1* and found that the deletion mutant was reduced in vegetative growth, conidial production, and sensitivity to various stresses. The Δ*ram1* mutant was also reduced in its virulence to plants due to defects in appressorium formation and invasive growth. Interestingly, the reduction of appressorium formation can be recovered by exogenous cAMP and IBMX. We further investigated that membrane localization of two RAS proteins, Ras1 and Ras2, was regulated by Ram1‐mediated farnesylation through their C‐terminus CaaX motifs. These findings indicate that farnesylation is involved in RAS protein‐mediated signalling pathways for appressorium formation and infection in *M. oryzae*.

## Results

### Characterization of farnesyltransferase β‐subunit Ram1 in *M. oryzae*

By using the *S. cerevisiae* Ram1 protein as a query, the farnesyltransferase β‐subunit Ram1 (MGG_01287T0) was identified through searching the *M. oryzae* genome database (Ensembl Fungi) (http://fungi.ensembl.org/Magnaporthe_oryzae/Info/Index). Phylogenetic tree analysis of Ram1 proteins was performed by using MEGA v. 5.10, which demonstrated that this protein is well conserved among eukaryotes. *Neurospora crassa* (EAA29571) and *Fusarium oxysporum* FOSC 3‐a (EWY88141.1) Ram1 are the closest matches to MoRam1 among the analysed organisms (Fig. S1, see Supporting Information). The conservation of Ram1 protein was also evaluated by multiple sequence alignment. The results showed that MoRam1 protein shares a 61% amino acid identity to that of *Colletotrichum graminicola*, 61% to *F. oxysporum*, 51% to *A. fumigatus*, 33% to *C. neoformans*, 34% to *S. cerevisiae*, 31% to *C. albicans*, 42% to *Caenorhabditis elegans* and 41% to *Homo sapiens* (Fig. S2, see Supporting Information) at the protein level with more than 65% query coverage.

### Expression of *RAM1* gene during development and infection process of *M. oryzae*

To determine the potential roles of *RAM1* in *M. oryzae*, we evaluated its transcription profile by quantitative real‐time PCR (qRT‐PCR). The results showed that, in mycelium, conidium, conidial germination, early formed appressorium and the late infection hypha, *RAM1* was highly expressed, while it was repressed in the early invasive hypha at 18 and 24 h post‐inoculation (hpi) (Fig. S3, see Supporting Information). This data suggested that expression of *RAM1* is fine‐tuned for development and infection.

To reveal the roles of farnesylation in *M. oryzae*, we subsequently attempted to obtain the deletion mutant of *RAM1* gene. The gene replacement construct was amplified by a split‐PCR strategy (Fig. S4A, see Supporting Information), which was subsequently transformed into the wild‐type strain for homologous recombination. After PCR‐mediated confirmation, we successfully obtained two independent Δ*ram1* deletion mutants with similar phenotypes (Fig. S4B, C, see Supporting Information). We randomly chose one mutant, KO4, for further analysis. The complement transformants were also generated by random insertion of the native *RAM1* gene into the Δ*ram1* mutant. All of the complement strains were recovered in phenotypic defects including growth and conidiation, suggesting that the phenotypic defects of the mutants resulted from *RAM1* disruption. We also chose one complement strain, termed cRAM1, for further analysis.

**Figure 1:**
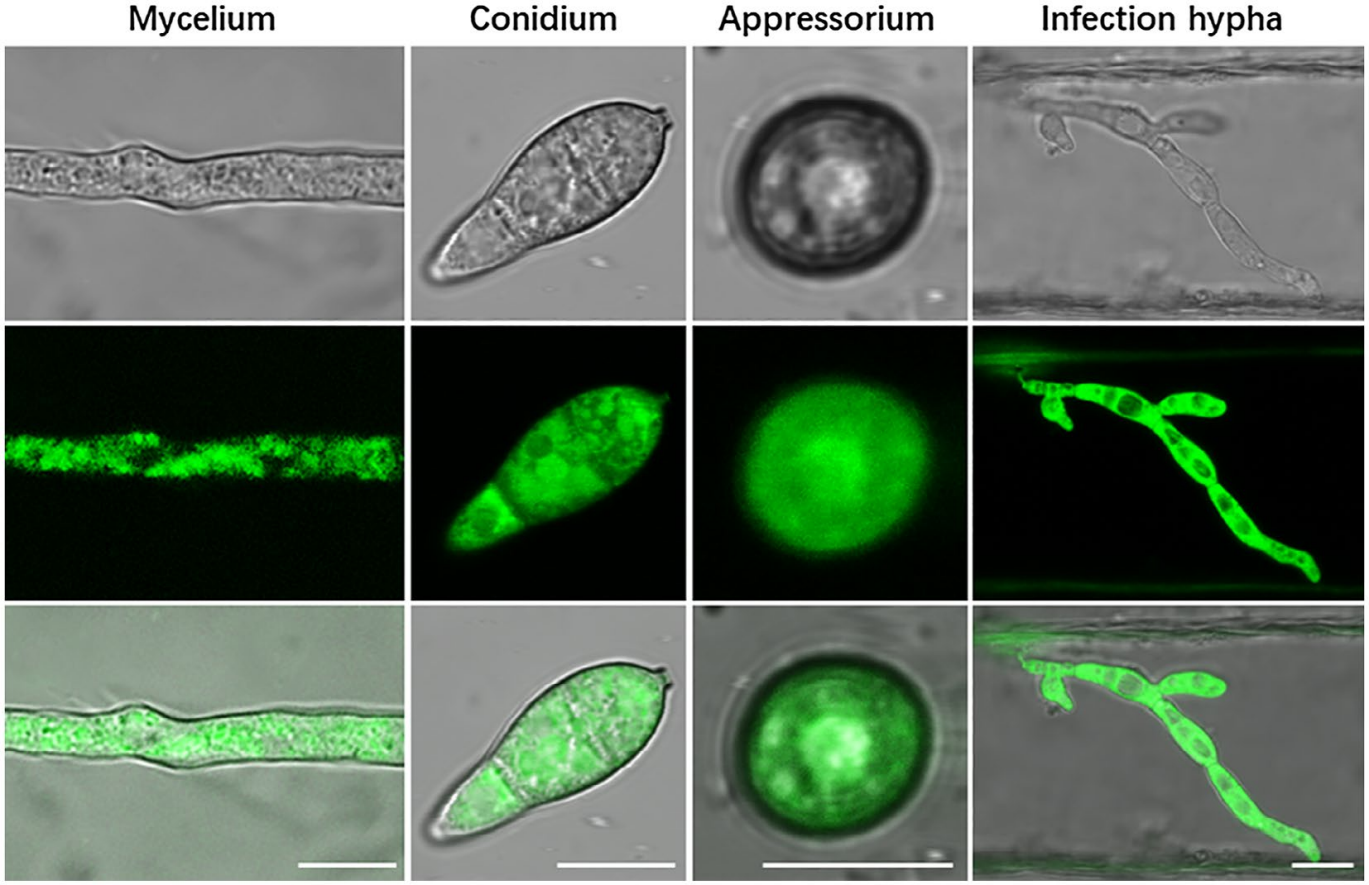
Subcellular localization of GFP‐tagged Ram1. The subcellular localization of Ram1 during mycelium, conidium, appressorium and infection hypha was observed by a confocal laser scanning microscope.

To investigate the subcellular localization of Ram1, the eGFP:Ram1 fusion construct was constructed and transformed into the Δ*ram1* mutant. After GFP signal detection and western blot analysis to confirm the integrity of GFP‐Ram1, one transformant, RAM1G, was obtained for further analysis. We found that the GFP‐Ram1 protein was highly expressed in all tested development stages, including mycelium, conidium, appressorium and infection hypha (Fig. 1). It seems from the results that Ram1 is mostly located in the cytoplasm at different tissues. These data suggest Ram1 may be required for all of the development and infection processes in *M. oryzae*.

### *RAM1* is required for fungal vegetative growth

**Figure 2:**
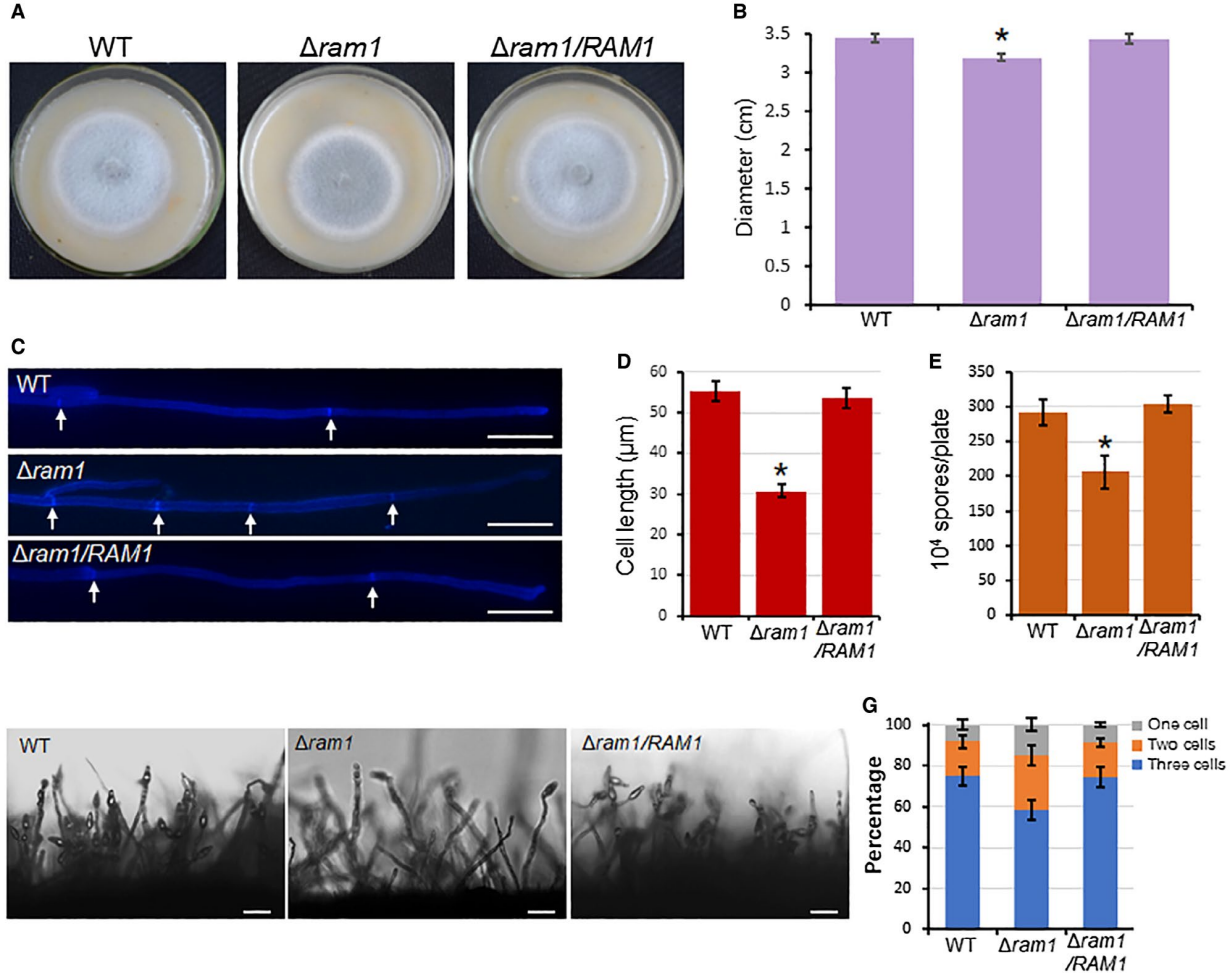
*RAM1* disruption affects vegetative growth and conidial production. (A) The Δ*ram1* mutant displays reduced colony growth. The indicated strains were cultured on oatmeal tomato agar (OTA) plates at 28 °C for 5 days. (B) Colony diameter. Significant differences compared with the wild‐type (WT) are indicated by an asterisk (*P* < 0.05). (C) Calcofluor White staining of hyphal tips shows the distance of septa. White arrows indicate the cell septa. Bar, 20 μm. (D) Average cell length of the hyphal tips. Significant differences compared with the wild‐type are indicated by an asterisk (*P* < 0.05). (E) Conidiation capacity. Conidia were collected from strains growing on OTA plates (Φ = 6 cm). Means and standard errors were calculated from three independent experiments (*n* > 100). Significant differences compared with the wild‐type are indicated by an asterisk (*P* < 0.05). (F) Conidiophore development was observed under light microscopy. Bars, 50 μm. (G) The percentages of different conidial morphologies. Means and standard errors were calculated from three independent experiments (*n* > 100).

To determine whether *RAM1* is related to vegetative growth in *M. oryzae*, the colony morphology of the Δ*ram1* mutant on oatmeal tomato agar plate (OTA) was observed. The colony size of Δ*ram1* was slightly reduced compared to the wild‐type at 120 hpi (Fig. 2A,B). The hyphal tip morphology of the Δ*ram1* mutant was then stained with Calcofluor White (CFW), and we found that the average length of apical hyphal cells was reduced compared to that of the wild‐type strain (Fig. 2C,D). These results indicate that *RAM1* is required for fungal vegetative growth.

### *RAM1* is important for conidium formation

Considering that the conidium is very important for the spread of the rice blast fungus, we also assessed the role of *RAM1* in conidium formation. First, the conidiation capacity of the Δ*ram1* mutant was measured. The result showed that conidia produced by the Δ*ram1* were 28% less than that of the wild‐type and the complementation strain (Fig. 2E).

The conidiophore formation of the Δ*ram1* mutant was also observed by using a light microscope. Under the conidiation condition, sparse conidia were formed on the conidiophores of the Δ*ram1* mutant, while dense conidia were formed on that of the wild‐type and complement strains (Fig. 2F). Also, we found that the cells number of spores in the Δ*ram1* mutant was abnormal compared to the wild‐type (Figs 2G and S5, see Supporting Information). For the wild‐type strain, around 75% of the conidia contained three cells, while for the Δ*ram1* mutant, only 58.3% of the conidia had three cells, 26% had two cells (with one septum) and 15% had one cell (without septum). This defect was recovered in the complementation strain cRAM1 (Fig. 2G). These data indicate that farnesylation is required for conidial septum formation.

### Deletion of *RAM1* leads to the attenuation of virulence

**Figure 3:**
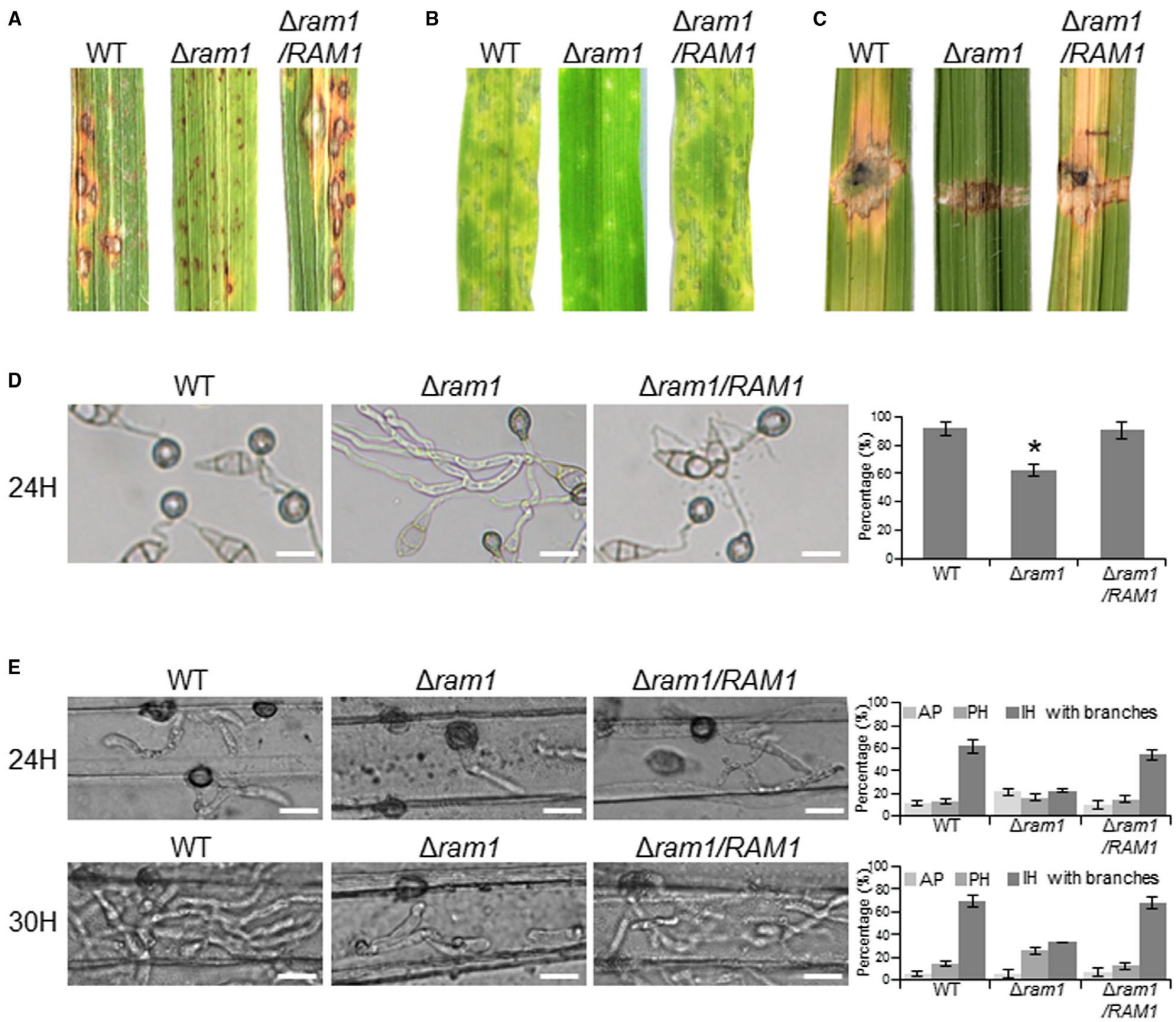
Deletion of *RAM1* leads to a reduction of virulence. (A) Lesions formed on rice leaves were infected by the indicated strains at 5 days post‐inoculation (dpi). (B) Lesions formed on barley leaves at 4 dpi. (C) Lesions formed on wounded rice leaves. (D) Appressorium formation of indicated strains on the hydrophobic surface at 24  h post‐inoculation (hpi). Significant differences compared with the wild‐type (WT) are indicated by an asterisk (*P* < 0.05). Bar, 20 μm. (E) Invasive hyphae formed in barley epidermal cells at 24 and 30 hpi. Bar, 20 μm. AP, appressoria; PH, primary hyphae, IH, invasive hyphae. Bar, 20 μm.

To determine whether deletion of *RAM1* affects the infection capacity, we tested the virulence of the wild‐type, Δ*ram1* mutant or cRAM1 strains on susceptible rice seedlings (*Oryzae sativa* cv. LTH). Conidial suspensions (1 × 10^5^ conidia/mL) of the above strains were sprayed onto rice at the 5‐leaf stage. The Δ*ram1* mutant showed an apparent reduction of lesion size and number compared to that of the other strains (Fig. 3A). One‐week‐old barley leaves (*Hordeum vulgare* cv. E9) were also inoculated by spraying a conidial suspension of those strains and a similar result was observed (Fig. 3B). We also inoculated the mycelial agar plugs onto the wounded rice leaves, which were scratched with a needle. We found that lesions caused by the Δ*ram1* mutant spread much less than that produced by the wild‐type and complement strains, indicating that invasive growth of the mutant was blocked (Fig. 3C). Based on these results, we conclude that Ram1‐mediated farnesylation is an important regulator of virulence during *M. oryzae* infection.

### Deletion of *RAM1* affects appressorium formation and invasive growth

To further understand why deletion of *RAM1* resulted in the reduction of virulence, we observed cellular infection processes. First, to determine the effect of *RAM1* deletion on appressorium formation, we observed the conidium germination process. Conidial suspension was inoculated on the hydrophobic coverslips, and the appressorium formation was observed at 24 hpi. At this time point, more than 90% of the wild‐type conidia can form appressoria, while it was only around 62% in the Δ*ram1* mutant, and many of the formed appressoria contained long germ tubes (Fig. 3D). This result indicates that *RAM1* plays a key role during germination and appressorium formation of *M. oryzae*. Second, we observed the infection process of the wild‐type, Δ*ram1* mutant and complement strains in barley epidermal cells. At 24 hpi, more than 70% of the wild‐type appressoria penetrated the plant cells, of which 61.6% developed branched invasive hyphae (IH). At this time point, the Δ*ram1* mutant just formed 16.8% primary IH and 22.8% one‐branched IH. At 30 hpi, around 69% of the wild‐type IH formed more than one branch, whereas it was only 35.8% in the Δ*ram1* mutant (Fig. 3E). Taken together, *RAM1* plays a key role in appressorium‐mediated penetration and invasive growth in *M. oryzae*.

### Deletion of *RAM1* resulted in increased sensitivity to various stresses

**Figure 4:**
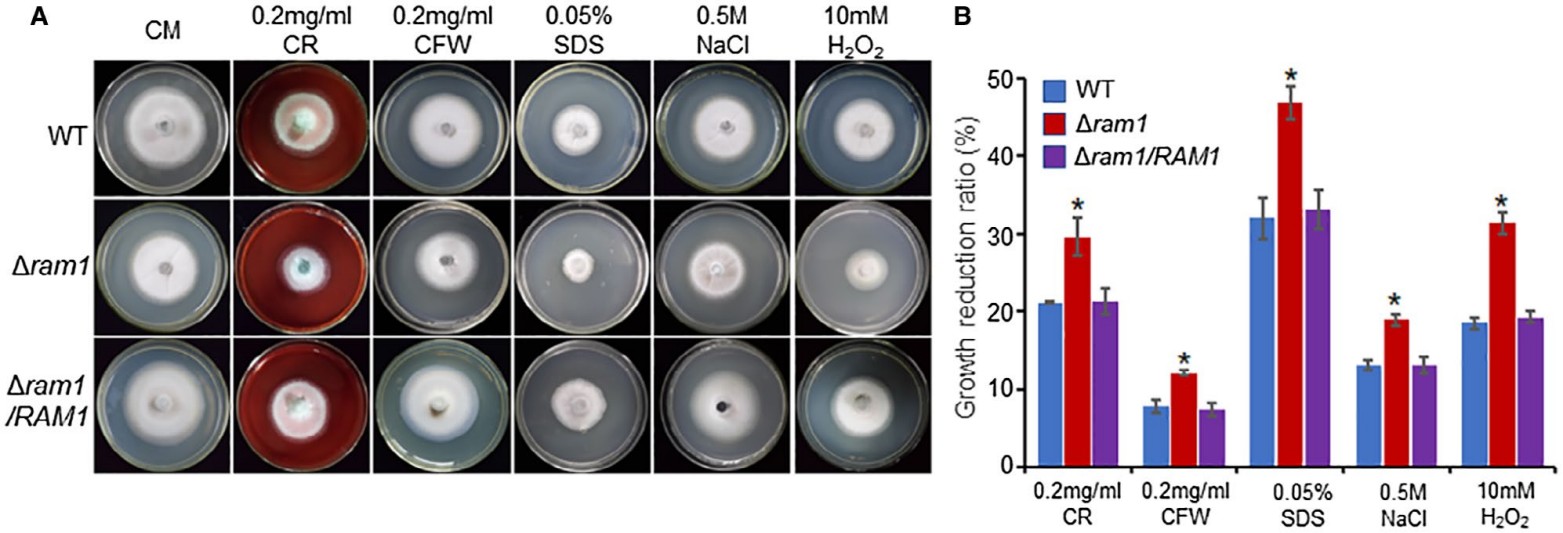
The deletion mutant of *RAM1* is sensitive to different stresses. (A) Colony morphology of indicated strains on complete medium (CM) plates supplemented with different indicated stress agents at 5 days post‐inoculation (dpi). (B) Statistical analysis of growth reduction rates of colony growth under different stresses. WT, wild‐type; CR, Congo Red; CFW, Calcofluor White; SDS, sodium dodecyl sulphate. Means and standard errors were calculated from three independent replicates. Significant differences are indicated by asterisks (*P* < 0.05).

To determine whether farnesylation is involved in stress response, we tested the sensitivity of the Δ*ram1* mutant to different stresses. The wild‐type, Δ*ram1* mutant, and complement strains were inoculated onto the complete medium (CM) plates supplemented with different reagents and grown for 120 h. The results show that the Δ*ram1* mutant is significantly sensitive to a series of stresses, especially the cell wall‐disturbing reagents (Fig. 4A,B). Under conditions of 0.1 mg/mL Calcofluor White (CFW), 0.2 mg/mL Congo Red (CR) or 0.005% sodium dodecyl sulphate (SDS), significant reduction of the colony growth happened as a result of the high sensitivity to these cell wall‐disturbing reagents, while the wild‐type and complementation strains were slightly affected (Fig. 4A,B). Increased sensitivity to other stresses, including osmotic stress (0.5 M NaCl) and oxidative stress (10 mM H_2_O_2_), was also observed (Fig. 4A,B). Interestingly, during the Δ*ram1* mutant infection, when we used an antioxidant NADPH oxidase inhibitor diphenylene iodonium (DPI, 0.5 μM) to treat the plant epidermis cells, the mutant's invasive growth defect was partially recovered (Fig. S6, see Supporting Information). These data suggest that Ram1‐mediated farnesylation is involved in responding to various stresses, including host cellular oxidative stress.

### RAM1 regulates cAMP pathway mediated appressorium formation

**Figure 5:**
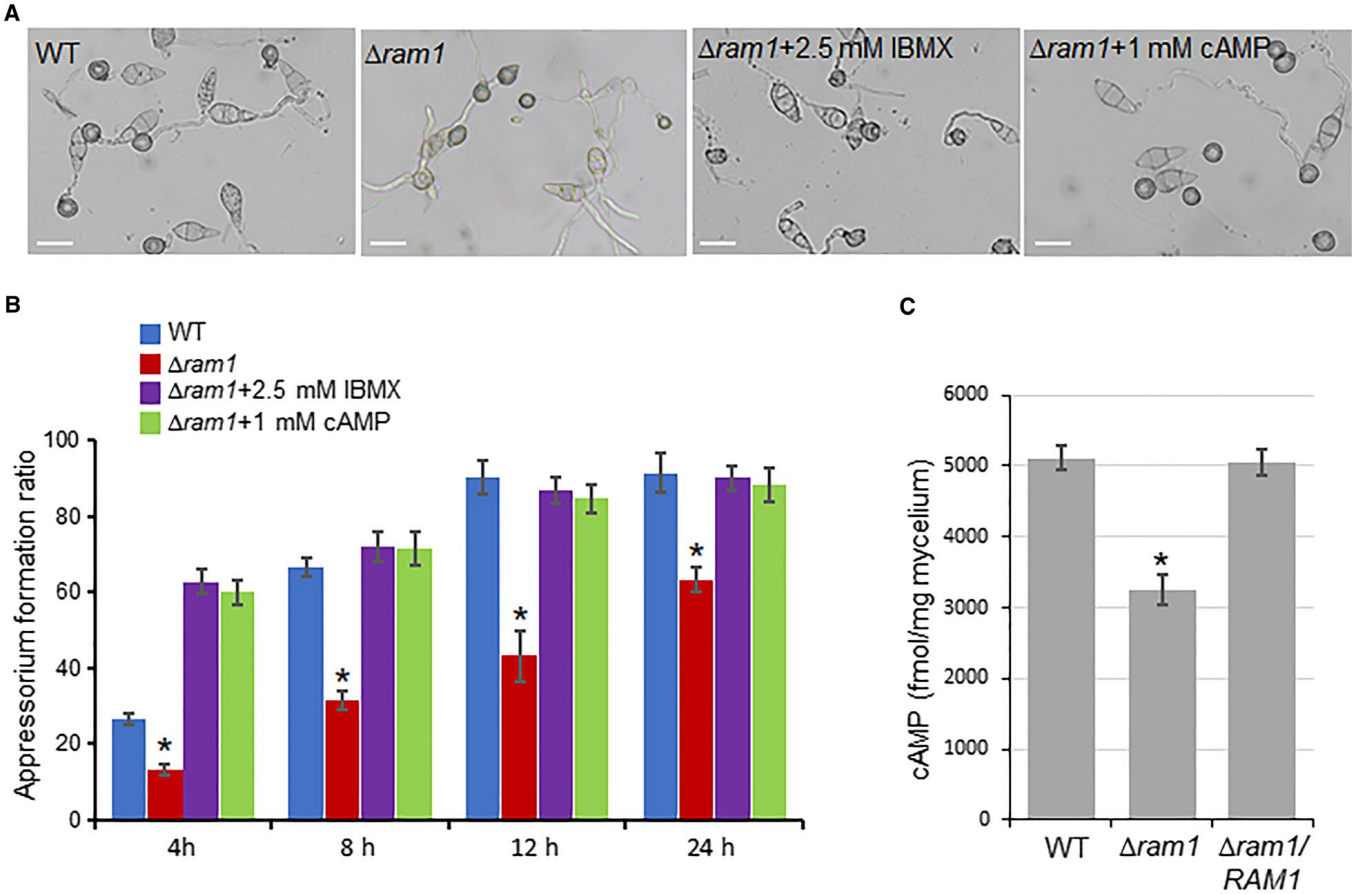
Exogenous addition of 3‐isobutyl‐1‐methylxanthine (IBMX) and cAMP recovers appressorium formation of the Δ*ram1* mutant on the hydrophobic surface. (A) Appressorium formation of the wild‐type (WT) strain and Δ*ram1* mutant induced by IBMX and cAMP. Conidial suspensions (1 × 10^6^ conidia/mL) from the WT or the Δ*ram1* mutant were assessed for appressorium formation in the presence of 2.5 mM IBMX and 1 mM cAMP. Bars, 20 μm. (B) Formation ratio of the wild‐type strain and Δ*ram1* mutant induced by IBMX and cAMP. Means and standard errors were calculated from three independent replicates. Asterisks indicate a statistically significant difference (*P* < 0.01). (C) Quantification of intracellular cAMP levels. The intracellular cAMP levels were detected in the mycelial stage. Two biological experiments with three replicates were assayed. Means and standard errors were calculated from three independent replicates. An asterisk indicates a statistically significant difference (*P* < 0.05).

In cells, the cAMP level is tightly regulated by adenylate cyclase and phosphodiesterase, enzymes responsible for synthesis and degradation, respectively (Sassone‐Corsi, 2012). For the Δ*ram1* mutant is defective in appressorium formation, and the cAMP signalling pathway is essential for appressorium formation in *M. oryzae*, we wondered if farnesylation acts upstream of the cAMP signalling pathway. We therefore added exogenous 8‐Br‐cAMP (a membrane permeable variant of cAMP) or IBMX (3‐isobutyl‐1‐methylxanthine, an inhibitor of cyclic AMP and cyclic GMP phosphodiesterases) (Lee and Dean, 1993; Mitchell and Dean, 1995) to enhance endogenous cAMP levels during appressorium formation of the Δ*ram1* mutant. An untreated wild‐type strain was used as a control. As shown in Fig. 5, when conidia of the Δ*ram1* mutant were treated with 1 mM cAMP or 2.5 mM IBMX, significant increases in the appressorium formation level were observed at 4, 8, 12 and 24 hpi. At 12 and 24 hpi, the appressorium formation ratio in the Δ*ram1* mutant treated with 1 mM cAMP or 2.5 mM IBMX rose to the level of the wild‐type strain (Fig. 5A,B). We also detected the endogenous cAMP level of the Δ*ram1* mutant in mycelium and found that it was significantly reduced compared with the wild‐type strain (Fig. 5C). These results support our speculation that Ram1‐mediated farnesylation plays a role in regulating the cAMP signalling pathway for appressorium formation in *M. oryzae*.

### *Magnaporthe oryzae* Ram1 can rescue defect of the *S. cerevisiae ram1* null mutant

**Figure 6:**
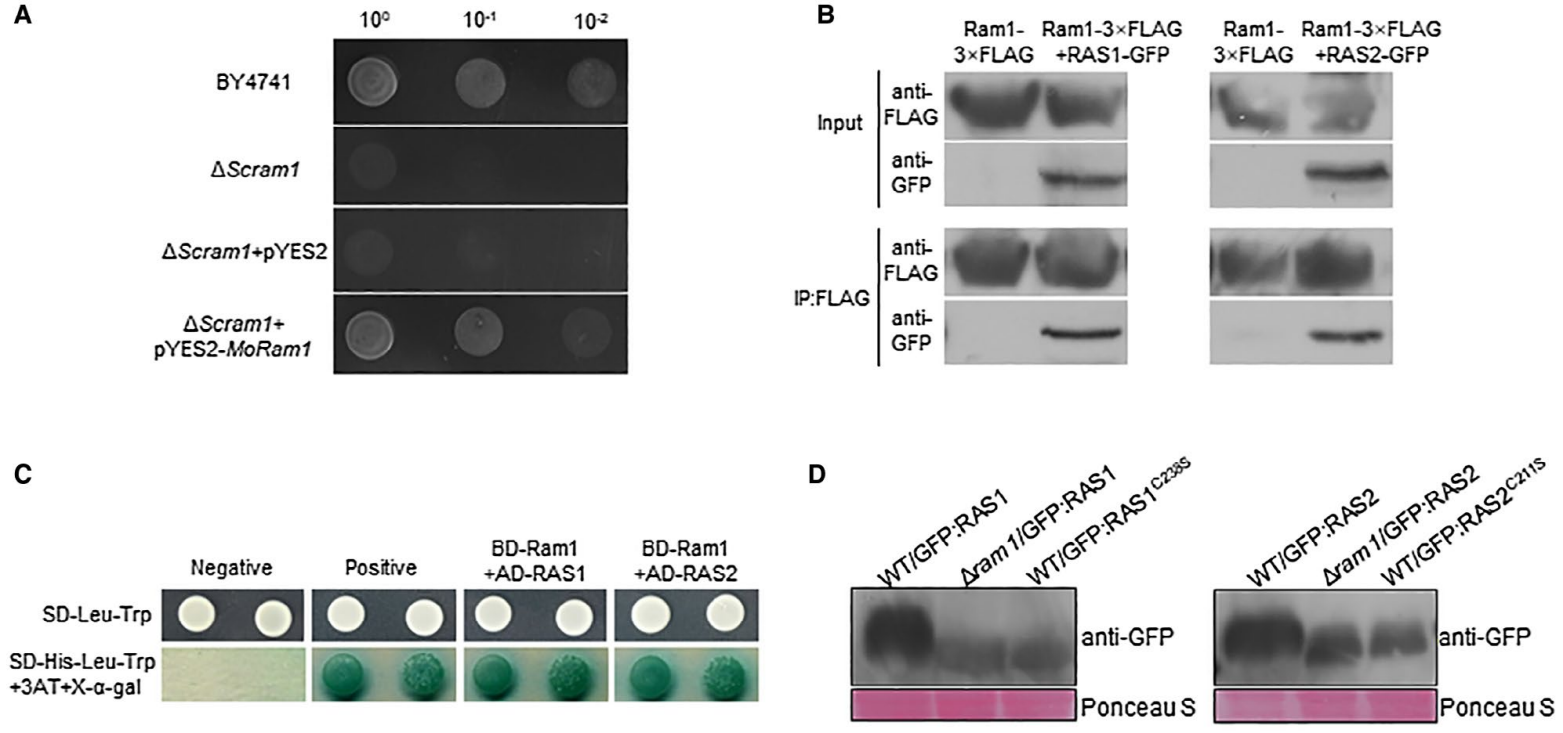
Ram1 regulates farnesylation of two RAS proteins. (A) Yeast complementation assay. Complementation of the *Saccharomyces cerevisiae ram1* mutant by *MoRAM1*. Cells of *S. cerevisiae ram1* and transformants of *ram1* carrying pYES2 or pYES2‐*MoRam1* were spotted in 10‐fold dilutions on synthetic defined (SD)‐Gal plates and incubated at 30 °C for 5 days. (B) Yeast two‐hybrid assay for the interaction between RAS1, RAS2 and Ram1. Yeast transformants expressing the prey and bait constructs were assayed for growth on SD‐Leu‐Trp and SD‐Leu‐Trp‐His plates and β‐galactosidase activities (LacZ). (C) Co‐immunoprecipitation (CoIP) analyses between RAS1, RAS2 and Ram1. The Ram1‐3xFLAG/RAS1‐GFP and Ram1‐3xFLAG/RAS2‐GFP were co‐expressed in the wild‐type (WT) strain. The Co‐IP experiment was performed with the anti‐FLAG beads, and the isolated protein was analysed by western blot using anti‐FLAG and anti‐GFP antibodies. (D) Protein level of RAS1 and RAS2 detected by western blot. Total proteins from extracts of indicated strains were separated by SDS‐PAGE and then subjected to western blot analysis with an anti‐GFP antibody. Ponceau S staining was used for evaluating loading levels.

To determine the molecular function of Ram1, we amplified the full‐length cDNA of Ram1 and ligated it into plasmid pYES2, which contained a galactose‐inducible promoter GAL1. The resulting construct, pYES2‐*Mo Ram1*, was introduced into the *S. cerevisiae ram1* null mutant, which was significantly defective for growth at 30 °C (He *et al.*, 1991). The resulting transformants were grown on yeast extract‐peptone (YP) medium with galactose (YPgal). The pYES2‐*Mo Ram1* transformant of the *S. cerevisiae ram1* null mutant recovered its growth at 30 °C. By contrast, the transformants carrying the empty vector pYES2 were defective in growth at 30 °C conditions, which is similar to the Δ*Scram1* mutant (Fig. 6A). Therefore, *M. oryzae* Ram1 can complement the *S. cerevisiae ram1* null mutant, suggesting that *M. oryzae* Ram1 also functions as a β‐subunit of the farnesyltransferase.

### RAS1 and RAS2 physically interact with Ram1

Ras proteins are small GTPases which can respond to external stimuli and activate various downstream signalling pathways for cellular responses (Zhou *et al.*, 2014) and whose subcellular localization is important for their functions (Prior and Hancock, 2012). Interestingly, Ras proteins were widely reported to be the farnesylation targets in eukaryotic cells, so we sought to determine if the functions of *M. oryzae* Ras proteins are regulated by farnesylation. Some Ras‐like proteins were found in *M. oryzae*, and they were used to perform farnesylation site prediction by GPS‐Lipid (http://lipid.biocuckoo.org/webserver.php) (Xie *et al.*, 2016). Six RAS‐like proteins, Ras1, Ras2, rho1, rho2, rho3, rho4 and ced‐10 (Fu *et al.*, 2018; Zheng *et al.*, 2007; Zhou *et al.*, 2014), were found to contain the C‐terminal CaaX motifs (Fig. S7, see Supporting Information), suggesting that they could be regulated by farnesylation. We chose Ras1 and Ras2, which have been reported to be putative regulatory factors of cAMP and PMK1‐MAPK signalling pathways in different fungi (Tamanoi, 2011; Zhou *et al.*, 2014), for validation.

We first confirmed the interactions between two RAS proteins and Ram1. The yeast two‐hybrid assay was performed first and demonstrated RAS1 and RAS2 indeed interact with Ram1 (Fig. 6B), suggesting a direct association between the two RAS proteins and Ram1. Co‐immunoprecipitation (Co‐IP) analysis was also employed to validate these interactions. The Ram1‐3 × FLAG and RAS1‐GFP fusion constructs were co‐transformed into protoplasts of strain P131. One of the resulting transformants, RAS1CO, was used for further analysis. Similarly, RAS2CO co‐expressing Ram1‐3 × FLAG and RAS2‐GFP fusion constructs were also obtained. Western blot analysis was performed with total proteins isolated from RAS1CO and RAS2CO, and the anti‐FLAG antibody detected a 59‐kDa band corresponding to Ram1‐3 × FLAG. Also, the anti‐GFP antibody detected 53‐ and 50‐kDa bands corresponding to RAS1‐GFP and RAS2‐GFP, respectively. Subsequently, in proteins eluted from anti‐FLAG M2 beads, 53‐kDa RAS1‐GFP and 50‐kDa RAS2‐GFP were detected with an anti‐GFP antibody in RAS1CO or RAS2CO, respectively (Fig. 6C). Transformants expressing the Ram1‐3 × FLAG construct were used as a negative control. All of the above indicate that two RAS proteins could interact with Ram1 in *M. oryzae*.

### RAM1 modify RAS proteins and regulate their plasma membrane localization

Western blotting analysis was used to further confirm the regulation of RAS1 and RAS2 by farnesylation. Western blot analysis showed noticeable changes in protein bands for both RAS1 and RAS2 proteins in the wild‐type, which indicates that changes in Ras protein migration when putative farnesylated Ras proteins had been compared to unfarnesylated Ras proteins in the Δ*ram1* mutant (Fig. 6D). In addition, both the GFP‐RAS1 and GFP‐RAS2 proteins in the Δ*ram1* mutant were less abundant than those in the wild‐type strain (Fig. 6D). Similarly, both of the GFP‐RAS1^C238S^ and GFP‐RAS2^C211S^ proteins in the wild‐type strain were present at comparable levels with the GFP‐RAS1 and GFP‐RAS2 proteins, respectively, in the Δ*ram1* mutant (Fig. 6D), revealing that C‐terminal modifications of RAS1 and RAS2 by farnesylation affect their protein abundance or stability.

**Figure 7:**
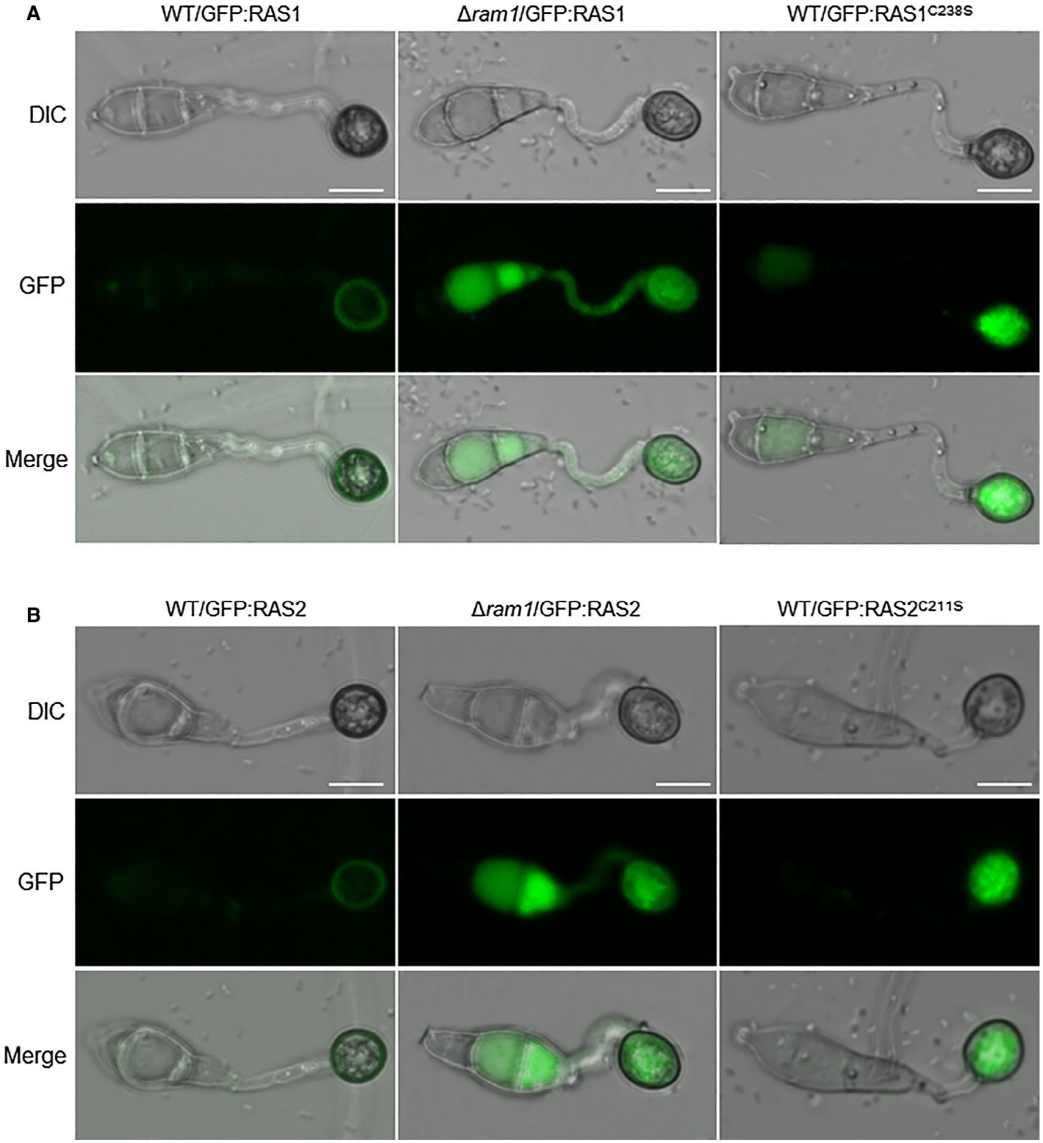
Farnesylation regulates plasma membrane localization of two RAS proteins. (A) Subcellular localization of RAS1 proteins in different strains. WT/GFP:RAS1, the wild‐type strain expressing eGFP:RAS1; Δ*ram1*/GFP:RAS1, the Δ*ram1* mutant expressing eGFP:RAS1; WT/GFP:RAS1^C238S^, the wild‐type strain expressing eGFP:RAS1^C238S^. (B) Subcellular localization of RAS2 proteins in different strains. WT/GFP:RAS2, the wild‐type strain expressing eGFP:RAS2; Δ*ram1*/GFP:RAS2, the Δ*ram1* mutant expressing eGFP:RAS2; WT/GFP:RAS2^C211S^, the wild‐type strain expressing eGFP:RAS1^C211S^. DIC, differential interference contrast. Bars, 10 μm.

To further determine whether Ram1 can regulate functions of RAS1 and RAS2 proteins, subcellular localization was observed. Both of the GFP‐RAS1 and GFP‐RAS2 proteins were uniformly distributed throughout the plasma membrane in appressorium of the wild‐type *M. oryzae*. In contrast, in the Δ*ram1* mutant background, both of the GFP‐RAS1 and GFP‐RAS2 proteins were distributed in the cytoplasm, but not in the plasma membrane (Fig. 7A,B), indicating that localization of both RAS proteins was regulated by Ram1. To assess the contributions of the C‐terminal cysteines in CaaX motifs in subcellular localization of RAS proteins, GFP‐RAS1 encoding C238S variant and GFP‐RAS2 encoding C211S variant were constructed and transformed into the wild‐type strain, respectively. Both of the GFP‐RAS1^C238S^ and GFP‐RAS2^C211S^ proteins were restricted to the cytoplasm in appressorium (Fig. 7A,B). We also detected subcellular localization of different types of RAS proteins in mycelium, conidium and infection hypha. Similarly, both of the GFP‐RAS1 and GFP‐RAS2 proteins can be well detected in the plasma membrane in the wild‐type strain, while the Δ*ram1* mutant background could be detected in the plasma membrane in all of the tested tissues. Both of the GFP‐RAS1^C238S^ and GFP‐RAS2^C211S^ proteins could not be well located in the plasma membrane (Figs S8A,B and S9A,B, see Supporting Information). These data confirm that farnesylation is essential for plasma membrane localization of RAS1 and RAS2. Altogether, Ram1‐mediated farnesylation directly regulates plasma membrane localization of RAS1 and RAS2.

### Farnesyltransferase inhibitor Tipifarnib suppresses appressorium formation and plasma membrane localization of RAS proteins

**Figure 8:**
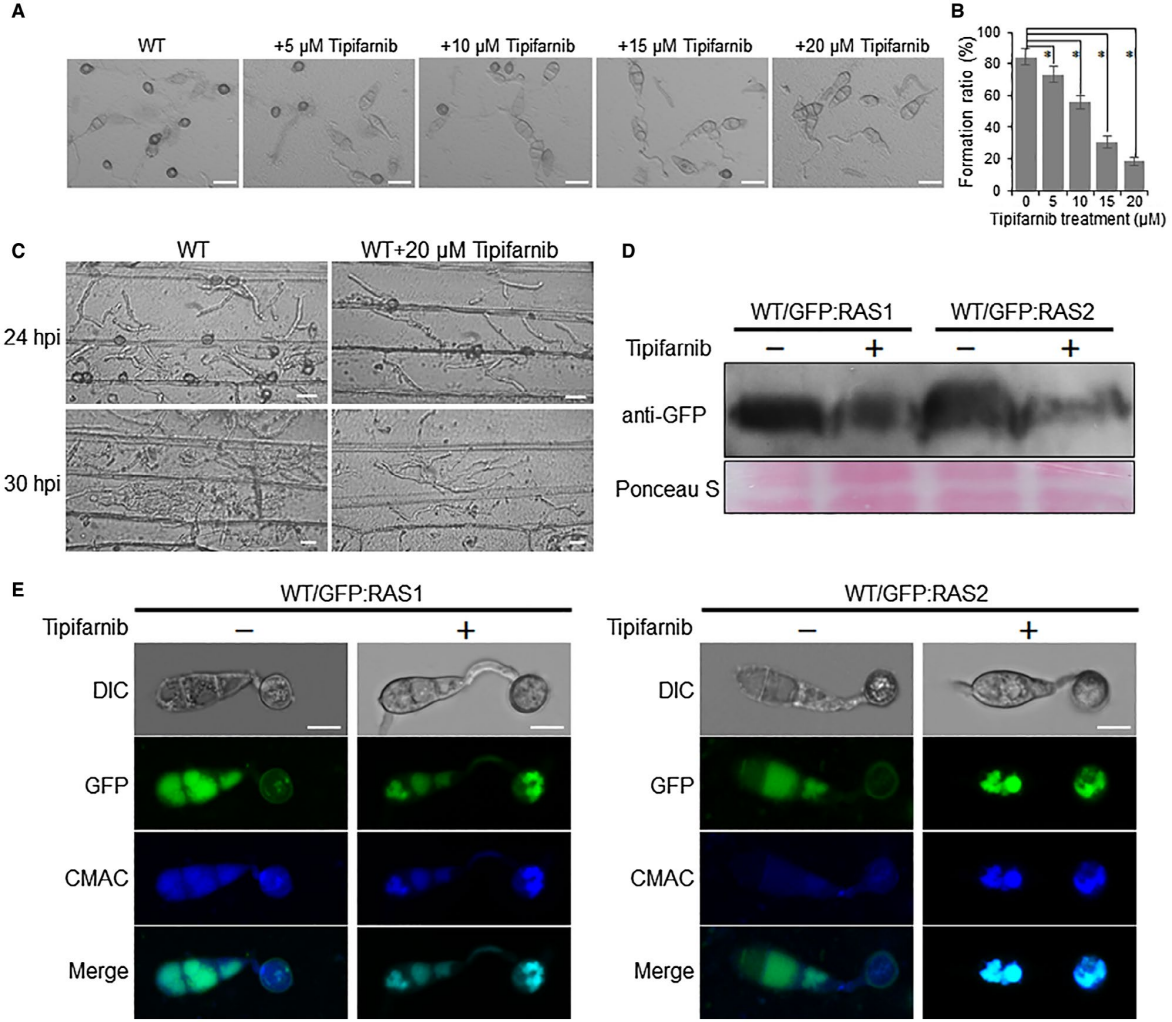
Farnesyltransferase inhibitor Tipifarnib suppress appressorium formation, invasive growth and localization of RAS proteins. (A) Tipifarnib inhibits appressorium of the wild‐type (WT) strain in a concentration‐dependent manner. Bars, 20 μm. (B) Appressorium formation ratios of the wild‐type strain treated with different concentrations of Tipifarnib at 12 h post‐inoculation (hpi). Means and standard errors were calculated from three independent replicates. Asterisks indicate a statistically significant difference (*P* < 0.05). (C) Effect of 20 μM Tipifarnib treatment on invasive growth of WT during infection in barley leaves at 24 and 30 hpi. Bars, 20 μm. (D) Protein levels of RAS proteins in the wild‐type strain with or without Tipifarnib treatment. Total proteins extracted from the mycelia cultured in liquid complete medium (CM) for 2 days, with or without 20 μM Tipifarnib, were used to perform Western blot with an anti‐GFP antibody. Ponceau S staining was used for evaluating loading levels. (E) Subcellular localization of RAS proteins in appressoria of the wild‐type strain with or without 20 μM Tipifarnib treatment. CMAC, 7‐amino‐4‐chloromethylcoumarin. Bars, 10 μm.

Inhibition of farnesyl transferase is the main step in restricting the farnesylation process (Bagchi *et al.*, 2018). We therefore used Tipifarnib, a competitive inhibitor of farnesyltransferase (FTase) (Lebowitz *et al.*, 2005), to test the effect of farnesylation block in *M. oryzae*. When we treated conidia of the wild‐type strain during appressorium formation with different concentrations of Tipifarnib (5, 10, 15 and 20 µM), a dramatic reduction in appressorium formation was observed in a dose‐dependent manner (Fig. 8A,B). At 12 hpi, the appressorium formation ratio of the wild‐type strain had decreased from more than 85% without Tipifarnib treatment to less than 20% with 20 µM Tipifarnib treatment (Fig. 8B). We also tested the effect of Tipifarnib on the invasive growth of *M. oryzae*. Tipifarnib with a concentration of 20 µM was added into the droplets of *M. oryzae* conidial suspension inoculated onto barley leaves at 18 hpi when the fungus has penetrated into the host cells. Cellular invasive growth was observed at 24 and 30 hpi. As shown in Fig. 8C, evident block in invasive hyphae growth were observed in Tipifarnib‐treated samples at both time points. Together, these data show that inhibition of farnesylation process blocks both appressorium formation and the invasive growth processes during infection of *M. oryzae*.

We also tested whether Tipifarnib can affect functions of the RAS proteins. Western blot analysis showed that in the wild‐type strain treated with 20 µM Tipifarnib both of the GFP‐RAS1 and GFP‐RAS2 proteins were less abundant than those without treatment (Fig. 8D), which was similar to the effects of *RAM1* deletion or farnesylation site mutations. As expected, GFP‐RAS1 and GFP‐RAS2 proteins were not normally distributed in the plasma membrane, but accumulated in an intracellular region, which was well co‐localized with the 7‐amino‐4‐chloromethylcoumarin (CMAC)‐stained vacuoles (Fig. 8E). We also treated invasive hyphae of the wild‐type strain by adding 20 µM Tipifarnib at 18 dpi, when the strain began to penetrate the host cell. The result demonstrates that although GFP‐RAS1 and GFP‐RAS2 proteins can also be detected in the plasma membrane, most of them accumulate in the intracellular regions (Fig. S9A,B, see Supporting Information). These results indicate that plasma membrane localization of the RAS proteins is blocked by the farnesyltransferase inhibitor Tipifarnib.

## Discussion

Farnesylation is a conserved post‐translational modification by which proteins can be modified at their C‐terminus CaaX motifs to allow subcellular localization (Maurer‐Stroh *et al.*, 2003; Zhang and Casey, 1996). Previous studies have revealed the importance of farnesylation in *S. cerevisiae*, *C. albicans*, *S. pombe*, *C. neoformans* and *A. fumigatus* (He *et al.*, 1991; Norton *et al.*, 2017; Vallim *et al.*, 2004; Yang *et al.*, 2000). However, only a few reports address the role of farnesylation in plant pathogenic fungi such as *U. maydis* and *U. hordei* (Caldwell *et al.*, 1995; Kosted *et al.*, 2000; Spellig *et al.*, 1994). In this study, we explain the function of FTase β‐subunit Ram1 to reveal general functions of farnesylation modification in the model plant pathogenic fungus *M. oryzae*.

Similar to the studies in other fungi, our results show that disruption of *M. oryzae* FTase β‐subunit *RAM1* resulted in significant phenotypic defects, including decreases in colony growth, conidiation, stress resistance and virulence. These data demonstrate a broad function of farnesylation in *M. oryzae*. We failed to obtain the disruption mutant of FTase α‐subunit gene *RAM2*, as found in *S. cerevisiae* and several human pathogenic fungi (He *et al.*, 1991; Song and White, 2003). This phenomenon reflected that FTase α‐subunit Ram2, which is also an α‐subunit of geranylgeranyltransferase type‐I complex (GGTase I), is essential in *M. oryzae* and it is also suggested that it plays an essential role in prenylation in *M. oryzae*.

Importantly, *RAM1* is required for the infection capacity of *M. oryzae*. We reasoned that the attenuated virulence in the *∆ram1* mutant resulted from two cellular mechanisms. First, the appressorium differentiation was significantly affected by the *∆ram1* mutant. Only around 60% conidia of the *∆ram1* mutant formed appressoria (Fig. 2E), suggesting that farnesylation plays regulatory roles in appressorium differentiation. Interestingly, the reduction of appressorium formation in the *∆ram1* mutant can be recovered by exogenous cAMP and IBMX (Fig. 5A,B). This result suggests that farnesylation functions upstream of the cAMP signalling pathway, which is found to be essential for appressorium formation. Second, the invasive growth in the host was also evidently blocked in the *∆ram1* mutant (Fig. 3E). This defect could be partly due to defected cell wall integrity and reduced stress resistance (Fig. 4A,B).

We also confirmed that the two Ras proteins, RAS1 and RAS2, are regulated by farnesylation. Ras proteins belong to a class of protein called small GTPase, which can switch between the active GTP and inactive GDP‐bound statuses. They can regulate cellular responses to external stimuli and mediate cellular signal transduction for cell growth, differentiation, and survival (Milburn *et al.*, 1990). Ras proteins have been proved to play a key role on the development and infection processes in different fungi (Bluhm *et al.*, 2007; Fortwendel *et al.*, 2004; Muller *et al.*, 2003; Waugh *et al.*, 2002). In *M. oryzae*, two Ras proteins, Ras1 and Ras2, were proved to interact with Mst50 and Mst11, two components of the Pmk1‐MAPK signalling pathway (Park *et al.*, 2006). MoRas2 was also shown to function in the upstream of both the cAMP signalling and Pmk1 MAPK pathways for appressorium morphogenesis in *M. oryzae* (Zhou *et al.*, 2014). It has been reported that RAS proteins are targets of farnesylation in different organisms. In this study, we identified some RAS proteins, including RAS1, RAS2, Rho1, Rho2, Rho3 and Rho4, contain farnesylation binding motif CaaX at their C‐terminus (Fig. S7, see Supporting Information). We subsequently confirmed that the protein levels and plasma membrane localization of RAS1 and RAS2 were directly regulated by farnesylation. In the *∆ram1* mutant, RAS1 and RAS2 were significantly reduced in protein levels (Fig. 6D), and either of them cannot be well located in the plasma membrane (Fig. 7A,B). Farnesylation site mutations in RAS1 and RAS2 also resulted in similar protein level reduction and plasma membrane mislocalization (Fig. 7). We noticed that mislocalization patterns of Ras1/Ras2‐GFP in the *∆ram1* background and the point site mutation mutants were different from the Tipifarnib treatment (Figs 6A,B and 7E). This phenomenon could be explained by the fact that Tipifarnib is a chemical that is harmful to the cell and induces large vacuole formation for drug degradation. At the same time, plasma membrane mislocalized Ras proteins could also be degraded in the vacuole. Considering that RAS1 and RAS2 are involved in both the cAMP signalling and Pmk1‐MAPK pathways, we propose that farnesylation‐regulated plasma membrane localization of RAS1 and RAS2 is vital for activating both appressorium formation signalling pathways. It is interesting to reveal the regulatory mechanisms of farnesylation on other targets, especially the remaining RAS proteins such as Rho1, Rho2, Rho3 and Rho4. Genomewide identification of FTase targets is also required.

In *M. oryzae*, it has been reported that regulators of G‐protein signalling (RGS proteins) negatively regulate heterotrimeric G‐protein cascades and control the conidiation and appressorium development, also probably via regulation of cAMP signalling (Liu *et al.*, 2007). In the Rgs1‐heterotrimeric G‐protein cascades, transmembrane protein Rgs1 may perceive extracellular signals to regulate the cAMP signalling pathway. Interestingly, Ras proteins are usually also plasma membrane proteins which can be activated by cell surface receptors to regulate downstream cellular processes, including RGS protein‐regulated events (Prior and Hancock, 2012). However, whether Ras proteins can regulate or work together with RGS proteins in *M. oryzae* requires further study.

As discussed above, disruption of *M. oryzae* FTase α‐subunit gene *RAM1* resulted in significant decreases in vegetative growth, conidiation capacity, stress resistance and virulence, while disruption of β‐subunit gene *RAM2* is lethal (data not shown). These results indicate that abolishing FTase activity could be an interesting target for antifungal drugs or fungicide development. As shown in our study, the FTase inhibitor Tipifarnib severely blocked both the appressorium formation and invasive growth of *M. oryzae* (Fig. 8A–C). Further analysis demonstrated that the farnesylation targets RAS1 and RAS2 were severely reduced in protein levels and mislocalized in the appressorium (Fig. 8D,E). For the Pmk1‐MAPK and cAMP signalling cascades, which play central roles in the infection‐related structure differentiation (Mitchell and Dean, 1995; Thines *et al.*, 2000; Xu and Hamer, 1996), inhibition of these two signalling pathways has been considered as an effective strategy to control fungal disease. Developing the fungal‐specific farnesyltransferase inhibitors should therefore also be an effective strategy for fungal disease control. It has been reported that farnesyltransferase inhibitors showed effective antifungal activity against the human pathogenic fungi, such as *Cryptococcus* (Hast MA *et al.*, 2011). Besides Tipifarnib, farnesyltransferase inhibitors, such as manumycin A, 2‐BP and FPT Inhibitor III, have been widely developed for controlling human cancers, which are commonly activated by RAS proteins (Agrawal and Somani, 2009; Appels *et al.*, 2005). In the future, identification of fungal‐specific farnesylation inhibitors might offer novel strategies to develop new fungicides.

Collectively, our findings support the fact that the Ram1‐mediated farnesylation process plays an important role in development, environmental response and pathogenesis in *M. oryzae*. These findings suggest that blocking the farnesylation process through FTases is a potential strategy to control plant fungal diseases.

## Experimental Procedures

### Strains and culture conditions

All the wild‐type strain of *M. oryzae* used in this study is P131 (Table S1, see Supporting Information) (Chen *et al.*, 2014). The fungal strains were grown on OTA medium at 28 °C. We incubated the mycelia in CM liquid culture at (180 rpm) 28 °C for 36 h for extracting gDNA, protein and protoplast isolation. Colony growth and conidiation were done as described by Chen *et al.* (2014). To evaluate the virulence and observe the infection process, conidia were harvested from 7‐day‐old OTA cultures. For testing stress sensitivity, strains were inoculated on CM plates supplemented with different stress agents (0.2 mg/mL Congo Red (CR), 0.1 mg/mL Calcofluor White (CFW), 0.005% sodium dodecyl sulphate (SDS), 0.5 M NaCl and 10 mM H_2_O_2_), and the colony diameters were measured 5 days post‐inoculation (dpi).

To observe the cell lengths of the hyphal tips, 10 μg/mL CFW (Sigma‐Aldrich, St. Louis, MO, USA) was used to stain hyphal cell walls and septa for 10 min in the dark, and the hyphal tips were observed under a fluorescence microscope (Ni90 microscope; Nikon, Tokyo, Japan) after being rinsed with phosphate‐buffered saline.

### Gene disruption and complementation

To generate the gene's replacement construct of *RAM1*, we amplified 1.5‐kb upstream and 1.5‐kb downstream of the gene's flanking sequences from the genomic DNA of the wild‐type strain. Both flanking sequences were fused with part of the hygromycin segment by overlap PCR. Subsequent PCR products were transformed into protoplasts of the wild‐type strain (Fig. S4, see Supporting Information). For complementation, a *RAM1* gene containing a 1.5‐kb promoter region and a 0.5‐kb terminator region was amplified and cloned into pKN plasmid (Wang *et al.*, 2018). The resulting construct pKN‐*RAM1 *(Table S2, see Supporting Information) was transformed into the Δ*ram1* mutant. CM plates supplemented with 250 μg/mL hygromycin B (Roche Diagnostics, Indianapolis, IN, USA) was used to select deletion transformants, with 400 μg/mL neomycin (Amresco, Solon, OH, USA) to select complementation transformants. PCR‐mediated methods were used to confirm different transformants. All the primers used in this study were shown in Table S3 (see Supporting Information).

### Subcellular localization

The eGFP:RAM1 fusion vector was generated by cloning the *RAM1* coding region into the C‐terminal of vector GFP gene in pKNRG, which contains the constitutive promoter RP27 fused (Wang *et al.*, 2018). The resulting plasmid pKNRG‐*RAM1* was transformed into the Δ*ram1* mutant and selected by 400 μg/mL neomycin. Similar strategies were applied to constructing plasmids of pKNRG‐*RAS1* and pKNRG‐*RAS2* (Table S2, see Supporting Information), respectively used for localization of RAS1 and RAS2 proteins. pKNRG‐*RAS1* and pKNRG‐*RAS2* were transformed into both the wild‐type strain and the Δ*ram1* mutant. The pKNRG‐*RAS1* and pKNRG‐*RAS2* plasmids were also used to generate CaaX motif mutation plasmids pKNRG‐*RAS1^C238S^* and pKNRG‐*RAS2^C211S^* (Table S2, see Supporting Information), which contain PCR‐mediated mutations from serine to cysteine at their farnesylation sites. All of the above strains were used to observe GFP fluorescence at different developmental stages and infection processes under a confocal microscope Leica TCS SP8 (Leica Microsystems, Mannheim, Baden‐Württemberg, Germany).

### Virulence test and infection process observation

Four‐week‐old rice seedlings (*O. sativa* cv. LTH) and 1‐week‐old barley leaves (*H. vulgare* cv. E9) were used to test the virulence of different fungal strains. The plants were sprayed by conidial suspensions with a concentration of 5 × 10^4^ conidia/mL in 0.025% Tween 20. After being incubated with full humidity at 28 °C for 5 days, the disease lesion was observed and photographed.

To observe appressorium formation, drops of conidial suspension (1 × 10^5^ conidia/mL) were inoculated onto a hydrophobic coverslip and incubated in a dark, moist chamber at 28 °C. The appressoria formation ratio was observed at 4, 8, 12 and 24 hpi and calculated using a microscope (Ni90; Nikon, Tokyo, Japan). For each test, three replicates were performed for each strain, with at least 100 conidia per replicate. To test the effect of cAMP and IBMX on appressorium formation of the Δ*ram1* mutant, 1 mM cAMP (Macklin Biochemical, Xuhui, Shanghai, China) and 2.5 mM IBMX (Solarbio, Tongzhou, Beijing, China) were added into the conidial suspension for inoculation. To test the effect of the farnesylation inhibitor Tipifarnib on appressorium formation, different concentrations (5, 10, 15 and 20 µM) of Tipifarnib (Medchem Express, Princeton, NJ, USA) were added into the conidial suspension for inoculation.

To observe the infection process in the host cells, the lower barley leaves were inoculated by conidial suspension (1 × 10^5^ conidia/mL) of different strains and incubated in a dark, moist chamber at 28 °C. Infection processes were observed by tearing down the lower barley epidermis for observation under a Nikon Ni90 microscope at 24 hpi and 30 hpi. The vacuoles of appressoria formed on the surface were stained with 10 µM CMAC (Thermo Fisher Scientific, Waltham, MA, USA) for 15 min, then the samples were observed after being washed. To test the effect of the farnesylation inhibitor Tipifarnib on invasive growth, 20 µM Tipifarnib was added into the conidial suspension at 18 hpi, and the invasive growth was observed at 24 hpi and 30 hpi. To evaluate the growth of IH in DPI‐treated barley cells, a conidial suspension supplemented with 0.5 mM DPI was dropped on barley leaves as previously reported (Chen *et al.*, 2014).

### Quantitative real‐time PCR analysis

To evaluate the expression level of *RAM1* at development stages and infection processes, different tissues were harvested. Mycelia were collected from cultures incubated in liquid CM for 48 h. Germ tubes and appressoria were collected on the hydrophobic surface at 3 and 12 hpi. We harvested invasive hyphae by tearing down the lower barley epidermis inoculated by conidia at 18, 24 and 42 hpi. Total RNA of these samples was extracted by using a TRIzol kit (Invitrogen, Carlsbad, CA, USA), and then used for preparing the cDNA templates. By using an SYBR Green PCR Master Mix kit (Takara, Dalian, China), qRT‐PCR was performed on an ABI 7500 real‐time PCR system (Applied Biosystems, Foster City, CA, USA).

### Quantification of endogenous cAMP

All strains in the study were cultured in liquid CM for 48 h. The mycelia were harvested and treated with liquid nitrogen, then lyophilized for 16 h. These samples were used to extract and quantify the cAMP levels as described previously (Liu *et al.*, 2007) using the cAMP Biotrak Immunoassay System (BD Biosciences, Franklin Lakes, NJ, USA).

### Yeast complementation

The *M. oryzae RAM1* cDNA was amplified and cloned into the vector pYES2. The resulting plasmid pYES2‐*Mo Ram1* was transformed into the yeast *ram1* null mutant. The subsequent transformants were selected on YP medium with galactose (YPGal) and grown on the SD medium at 30 °C for 5 days.

### Yeast two‐hybrid assays

The bait construct was generated by cloning *RAM1* into pGBKT7. The prey constructs were generated by cloning RAS1 and RAS2 into pGADT7. The prey and bait constructs pairs BD‐Ram1/AD‐RAS1 and BD‐Ram1/AD‐RAS2 were co‐transformed into the yeast strain AH109 according to the manufacturer's instructions (Clontech, San Francisco, CA, USA). The transformants from SD‐Trp‐Leu plates were isolated and used to grow on SD‐Trp‐Leu‐His and SD‐Trp‐Leu‐His‐Ade media. The positive and negative control strains were obtained from the BD library construction and screening kit (Clontech, San Francisco, CA, USA).

### Co‐immunoprecipitation (Co‐IP) assay

To confirm the interaction between two RAS proteins and Ram1 *in vivo*, the coding regions for RAS1 or RAS2, respectively, were cloned into pKNRG, and the resulting constructs were pKNRG‐RAS1 and pKNRG‐RAS2. The coding region for Ram1 was cloned into pKNFLAG to gain the pKNFLAG‐Ram1. The pKNRG‐RAS1/pKNFLAG‐Ram1 and pKNRG‐RAS2/pKNFLAG‐Ram1 were co‐transformed into protoplasts of strain P131. To perform the Co‐IP assay, total proteins were extracted from the resulting transformants and then incubated with the anti‐FLAG M2 affinity resins (Sigma‐Aldrich, St. Louis, MO, USA). Proteins eluted from the M2 resins were analysed by western blot with the anti‐FLAG and anti‐GFP antibodies (​Abmart, Xuhui, Shanghai, China).

### Western blotting

The eGPF fused *RAS1*, *RAS1^C238S^*, *RAS2* and *RAS2^C211S^* constructs were transformed into the wild‐type or Δ*ram1* mutants, respectively. To extract total proteins, around 0.2 g mycelia of each transformant was ground into powder using liquid nitrogen and resuspended in 1 mL of extraction buffer with 1 mM PMSF (Sigma‐Aldrich, St. Louis, MO, USA). Total proteins were separated on a 12% SDS‐PAGE gel and used for western blot analysis with an anti‐GFP as the primary antibody (1:5000, Abmart) and anti‐rabbit horseradish peroxidase as the secondary antibodies (1:10000) (Abmart, Xuhui, Shanghai, China). The results were detected by an enhanced chemiluminescence detection system (Amersham Biosciences, Piscataway, NJ, USA). To determine the effect of the farnesyltransferase inhibitor, the mycelia of these transformants were treated with 20 µM Tipifarnib before protein extraction.

## Supporting information


**Fig. S1** Phylogenetic tree analyses of RAM1 proteins. Numbers at nodes represent the percentages of occurrence, and the scale bar represents the number of amino acid differences per site. GenBank accession numbers are followed by species.

**Fig. S2** Alignment of the amino acid sequences of RAM1. Amino acid sequences were obtained by BLAST and aligned with CLUSTAL W (http://www.ch.embnet.org/software/ClustalW.html). Identical and similar residues are indicated by colour characters. Sequences aligned were the predicted products of *Magnaporthe oryzae* RAM1 (EHA54398.1) and RAM1 orthologues from *Saccharomyces cerevisiae* (CAA98656.1), *Candida albicans* (AOW29142.1), *Cryptococcus neoformans* (AAN87033.1), *Aspergillus fumigatus* (KEY75842.1), *Colletotrichum graminicola* M1.001 (XP_008099870.1), *Fusarium oxysporum* f. sp. *lycopersici* (XP_018236816.1), *Arabidopsis thaliana* (OAP01011.1), *Caenorhabditis elegans* (NP_506580.1) and *Homo sapiens *(NP_002019.1).

**Fig. S3** Phase‐specific expression of *RAM1*. The phase‐specific expression of *RAM1* was quantified by quantitative real‐time PCR with a synthesis of cDNA from each sample, including mycelia, conidia, germ tubes, appressoria and invasive hyphae at indicated time points. Relative abundance was normalized by *MoTub1*. Three independent biological experiments using three replicates in each were performed. HY, mycelial hyphae; CO, conidia; AP, appressoria; IH, invasive hyphae.

**Fig. S4** Replacement of *Magnaporthe oryzae* *RAM1*. (A) Gene replacement of *RAM1* through a split‐marker approach. White bars represent genomic regions upstream and downstream of the *RAM1 *coding sequence that was amplified and fused to segments of the hygromycin phosphotransferase (HYG) cassette. (B) PCR verification of the flanking sequences besides the replacement fragment by using primer pairs of LCK/HCK‐up and RCK/HCK‐down. (C) PCR verification by amplifying the *RAM1* gene in the transformants and the wild‐type strain (WT).

**Fig. S5** Conidial septa of the *RAM1* deletion mutants stained with Calcofluor White (CFW).

**Fig. S6** Invasive growth of invasive hyphae restored by diphenylene iodonium (DPI) treatment. Barley leaves were treated with or without DPI (0.5 mM) dissolved in DMSO. Invasive growth was observed at 30 hpi. Bars, 20 μm.

**Fig. S7** Farnesylation site prediction in the C‐terminal of the RAS proteins in *Magnaporthe oryzae*. The farnesylation sites were predicted by GPS‐Lipid (http://lipid.biocuckoo.org/webserver.php) (Xie *et al.*, 2016). The CaaX motifs are indicated in blue and the farnesylation sites are indicated in red.

**Fig. S8** Subcellular localization of RAS proteins in mycelium and conidium. (A) Subcellular localization of RAS1 and RAS2 in mycelium. (B) Subcellular localization of RAS1 and RAS2 in conidium. WT/GFP:RAS1, the wild‐type strain expressing eGFP:RAS1; Δ*ram1*/GFP:RAS1, the Δ*ram1* mutant expressing eGFP:RAS1; WT/GFP:RAS1^C238S^, the wild‐type strain expressing eGFP:RAS1^C238S^; WT/GFP:RAS2, the wild‐type strain expressing eGFP:RAS2; Δ*ram1*/GFP:RAS2, the Δ*ram1* mutant expressing eGFP:RAS2; WT/GFP:RAS2^C211S^, the wild‐type strain expressing eGFP:RAS1^C211S^.

**Fig. S9** Subcellular localization of RAS proteins in invasive hypha. WT/GFP:RAS1, the wild‐type strain expressing eGFP:RAS1; Δ*ram1*/GFP:RAS1, the Δ*ram1* mutant expressing eGFP:RAS1; WT/GFP:RAS1^C238S^, the wild‐type strain expressing eGFP:RAS1^C238S^. WT/GFP:RAS2, the wild‐type strain expressing eGFP:RAS2; Δ*ram1*/GFP:RAS2, the Δ*ram1* mutant expressing eGFP:RAS2; WT/GFP:RAS2^C211S^, the wild‐type strain expressing eGFP:RAS1^C211S^. Bars, 10 μm.

**Table S1** Fungal strains used in this study.

**Table S2** Plasmids used in this study.

**Table S3** Primers used in this study.
