# Supplementary material for: The farnesyltransferase β‐subunit RAM1 regulates localization of RAS proteins and appressorium‐mediated infection in Magnaporthe oryzae
Source: Mol Plant Pathol. 2019 Jun 27;20(9):1264–78. doi: 10.1111/mpp.12838 (PMC6715606; doi:10.1111/mpp.12838)
Supplement: Supplementary file 1 — Fig. S1 Phylogenetic tree analyses of RAM1 proteins. Numbers at nodes represent the percentages of occurrence, and the scale bar represents the number of amino acid differences per site. GenBank accession numbers are followed by species. [file MPP-20-1264-s001.doc]

**
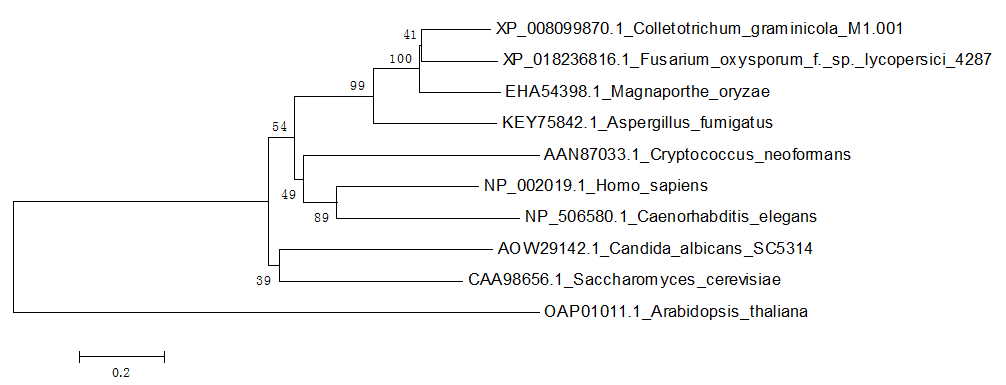
**

**Fig. S1 Phylogenetic tree analyses of RAM1 proteins.** Numbers at nodes represent the percentages of occurrence, and the scale bar represents the number of amino acid differences per site. GenBank accession numbers are followed by species.
