# Supplementary material for: The farnesyltransferase β‐subunit RAM1 regulates localization of RAS proteins and appressorium‐mediated infection in Magnaporthe oryzae
Source: Mol Plant Pathol. 2019 Jun 27;20(9):1264–78. doi: 10.1111/mpp.12838 (PMC6715606; doi:10.1111/mpp.12838)
Supplement: Supplementary file 2 — Fig. S2 Alignment of the amino acid sequences of RAM1. Amino acid sequences were obtained by BLAST and aligned with CLUSTAL W (http://www.ch.embnet.org/software/ClustalW.html). Identical and similar residues are indicated by colour characters. Sequences aligned were the predicted products of Magnaporthe oryzae RAM1 (EHA54398.1) and RAM1 orthologues from Saccharomyces cerevisiae (CAA98656.1), Candida albicans (AOW29142.1), Cryptococcus neoformans (AAN87033.1), Aspergillus fumigatus (KEY75842.1), Colletotrichum graminicola M1.001 (XP_008099870.1), Fusarium oxysporum f. sp. lycopersici (XP_018236816.1), Arabidopsis thaliana (OAP01011.1), Caenorhabditis elegans (NP_506580.1) and Homo sapiens (NP_002019.1). [file MPP-20-1264-s002.doc]

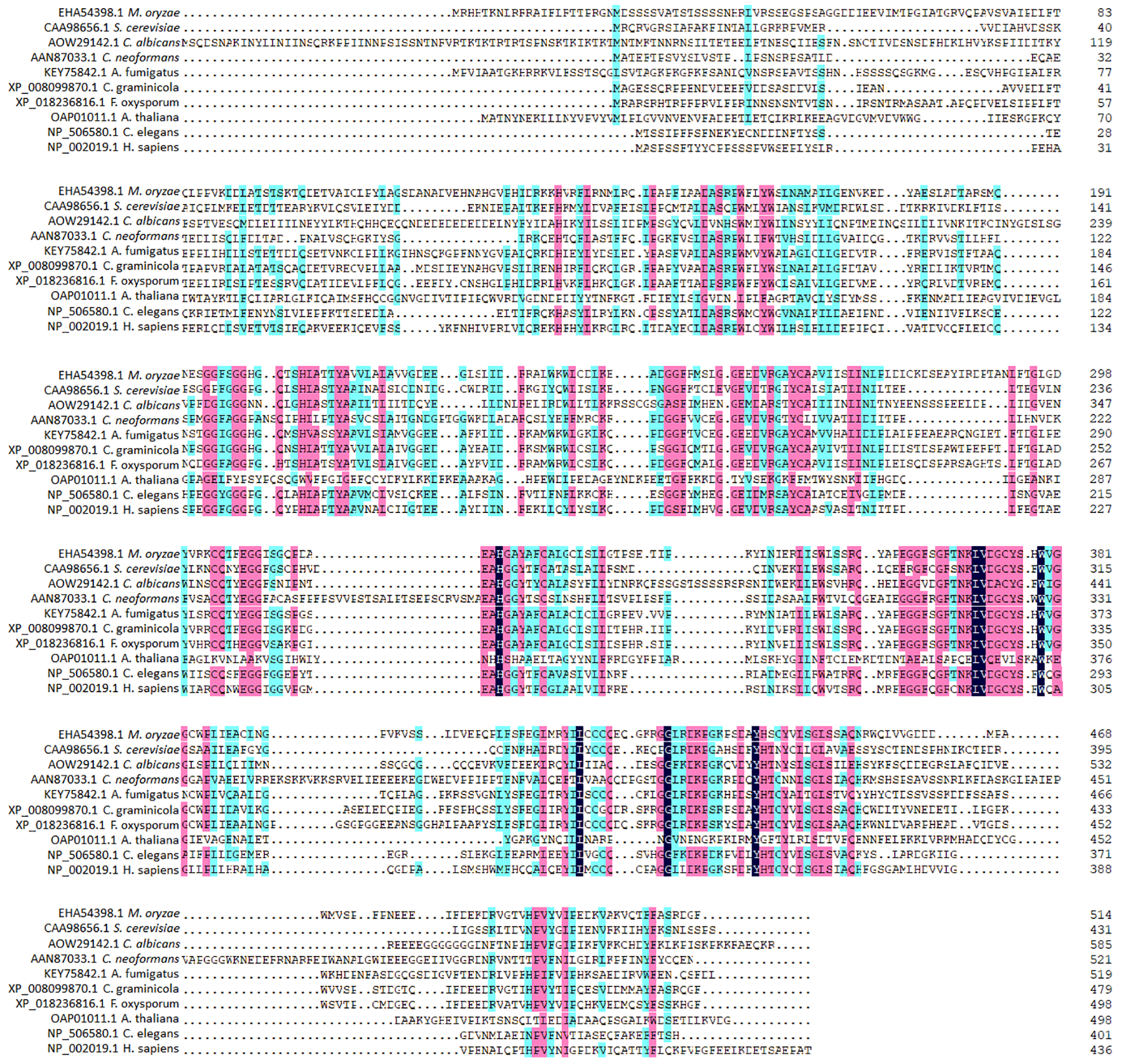


**Fig. S2 Alignment of the amino acid sequences of RAM1.** Amino acid sequences were obtained by BLAST and aligned with CLUSTAL W (http://www.ch.embnet.org/software/ClustalW.html). Identical and similar residues are indicated by color characters. Sequences aligned were the predicted products of *M. oryzae* RAM1 (EHA54398.1), and RAM1 orthologues from *Saccharomyces cerevisiae* (CAA98656.1), *Candida albicans* (AOW29142.1), *Cryptococcus neoformans* (AAN87033.1), *Aspergillus fumigatus* (KEY75842.1), *Colletotrichum graminicola* M1.001 (XP_008099870.1), *Fusarium oxysporum f. sp. lycopersici* (XP_018236816.1), *Arabidopsis thaliana* (OAP01011.1) , *Caenorhabditis elegans* (NP_506580.1) and *Homo sapiens* (NP_002019.1).
