# Supplementary material for: The farnesyltransferase β‐subunit RAM1 regulates localization of RAS proteins and appressorium‐mediated infection in Magnaporthe oryzae
Source: Mol Plant Pathol. 2019 Jun 27;20(9):1264–78. doi: 10.1111/mpp.12838 (PMC6715606; doi:10.1111/mpp.12838)
Supplement: Supplementary file 3 — Fig. S3 Phase‐specific expression of RAM1. The phase‐specific expression of RAM1 was quantified by quantitative real‐time PCR with a synthesis of cDNA from each sample, including mycelia, conidia, germ tubes, appressoria and invasive hyphae at indicated time points. Relative abundance was normalized by MoTub1. Three independent biological experiments using three replicates in each were performed. HY, mycelial hyphae; CO, conidia; AP, appressoria; IH, invasive hyphae. [file MPP-20-1264-s003.doc]

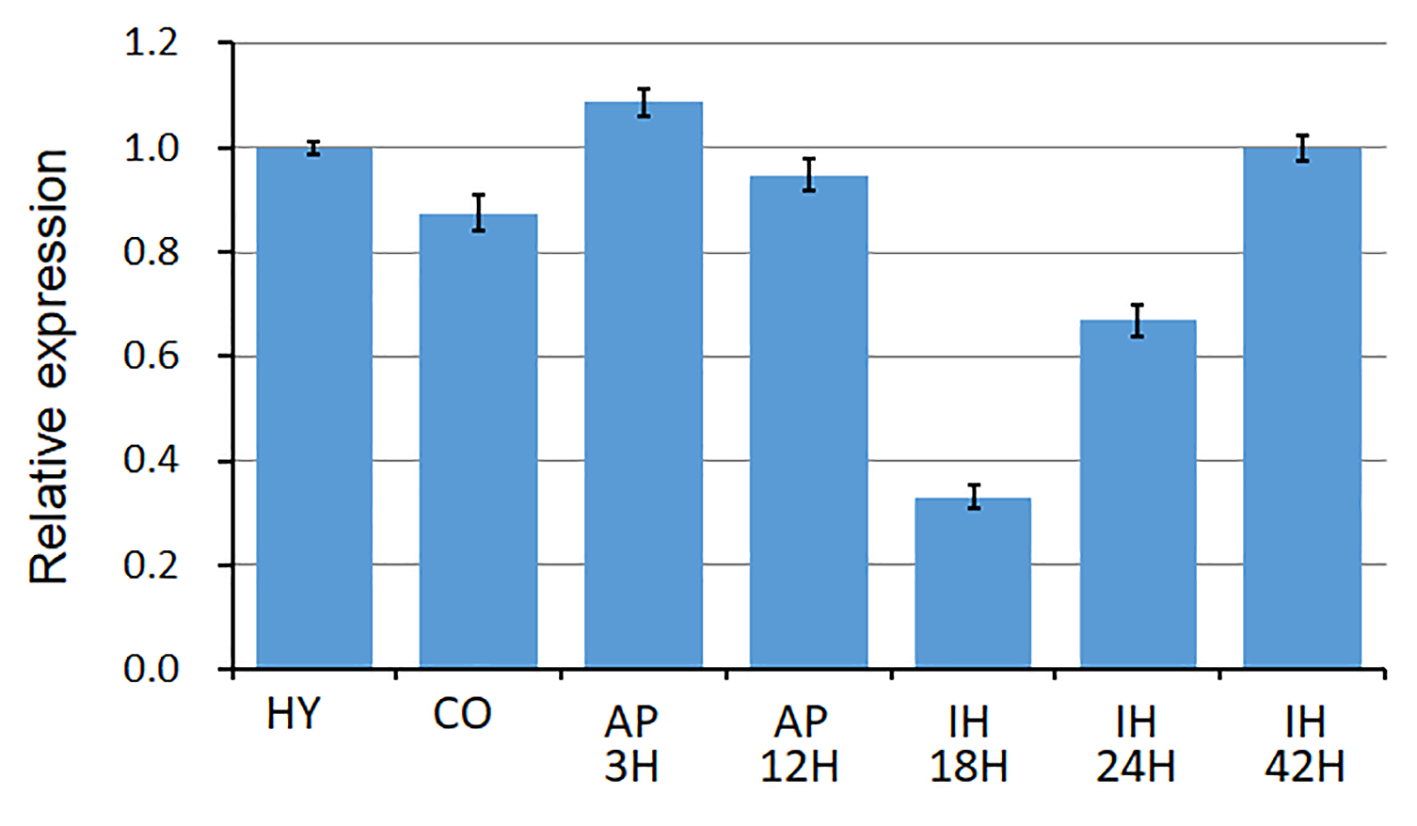


**Fig. S3 Phase specific expression of *RAM1*.** The phase specific expression of *RAM1* was quantified by quantitative real-time PCR with synthesis of cDNA from each sample including mycelia, conidia, germ tubes, appressoria and infection hyphae at indicated time points. Relative abundance was normalized by *MoTub1*. Three independent biological experiments with three replicates in each were performed. HY : Mycelial hyphae; CO: Conidia; AP : Appressoria ; IH : infection hyphae.
