# Supplementary material for: The farnesyltransferase β‐subunit RAM1 regulates localization of RAS proteins and appressorium‐mediated infection in Magnaporthe oryzae
Source: Mol Plant Pathol. 2019 Jun 27;20(9):1264–78. doi: 10.1111/mpp.12838 (PMC6715606; doi:10.1111/mpp.12838)
Supplement: Supplementary file 4 — Fig. S4 Replacement of Magnaporthe oryzae RAM1. (A) Gene replacement of RAM1 through a split‐marker approach. White bars represent genomic regions upstream and downstream of the RAM1 coding sequence that was amplified and fused to segments of the hygromycin phosphotransferase (HYG) cassette. (B) PCR verification of the flanking sequences besides the replacement fragment by using primer pairs of LCK/HCK‐up and RCK/HCK‐down. (C) PCR verification by amplifying the RAM1 gene in the transformants and the wild‐type strain (WT). [file MPP-20-1264-s004.doc]

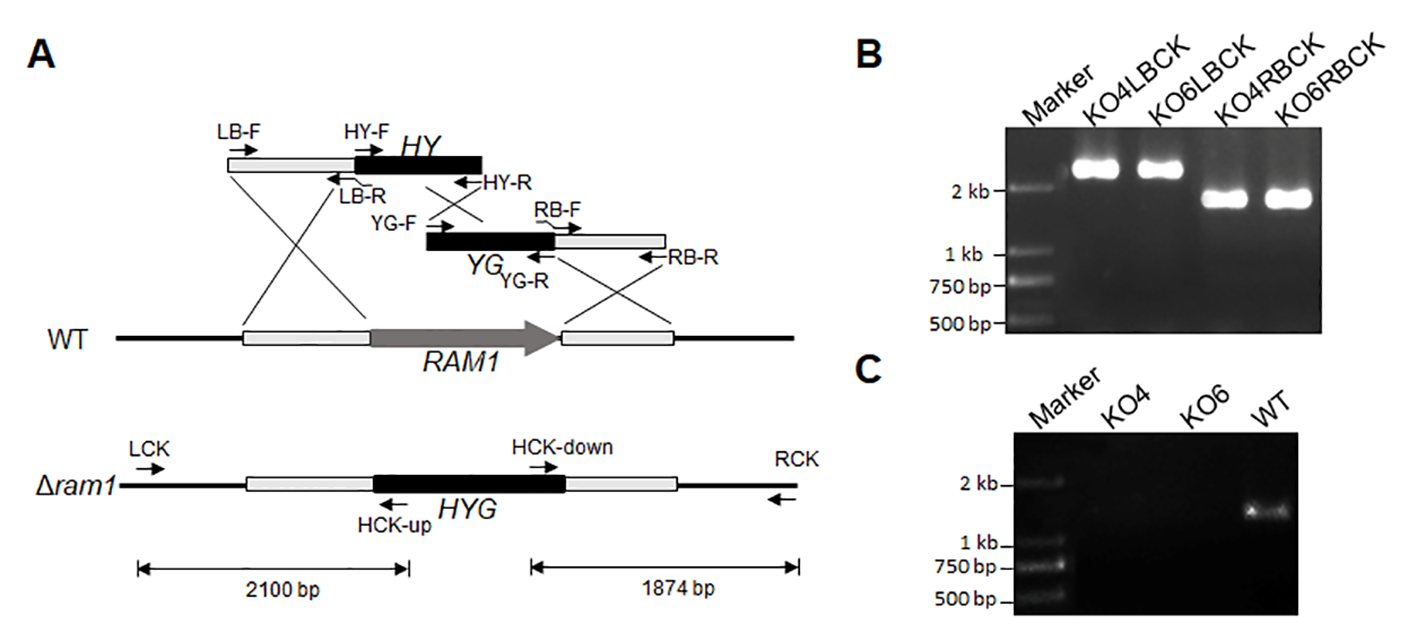


**Fig. S4 Replacement of *M. oryzae* *RAM1*.** (A) Gene replacement of *RAM1* through a split-marker approach. White bars represent genomic regions upstream and downstream of the *RAM1* coding sequence that were amplified and fused to segments of the hygromycin phosphotransferase (HYG) cassette. (B) PCR verification of the flanking sequences beside the replacement fragment by using primer pairs of LCK/HCK-up and RCK/HCK-down. (C) PCR verification by amplifying the *RAM1* gene in the transformants and the wild-type strain (WT).
