# Supplementary figures and images for: The farnesyltransferase β‐subunit RAM1 regulates localization of RAS proteins and appressorium‐mediated infection in Magnaporthe oryzae
Source: Mol Plant Pathol. 2019 Jun 27;20(9):1264–78. doi: 10.1111/mpp.12838 (PMC6715606; doi:10.1111/mpp.12838)

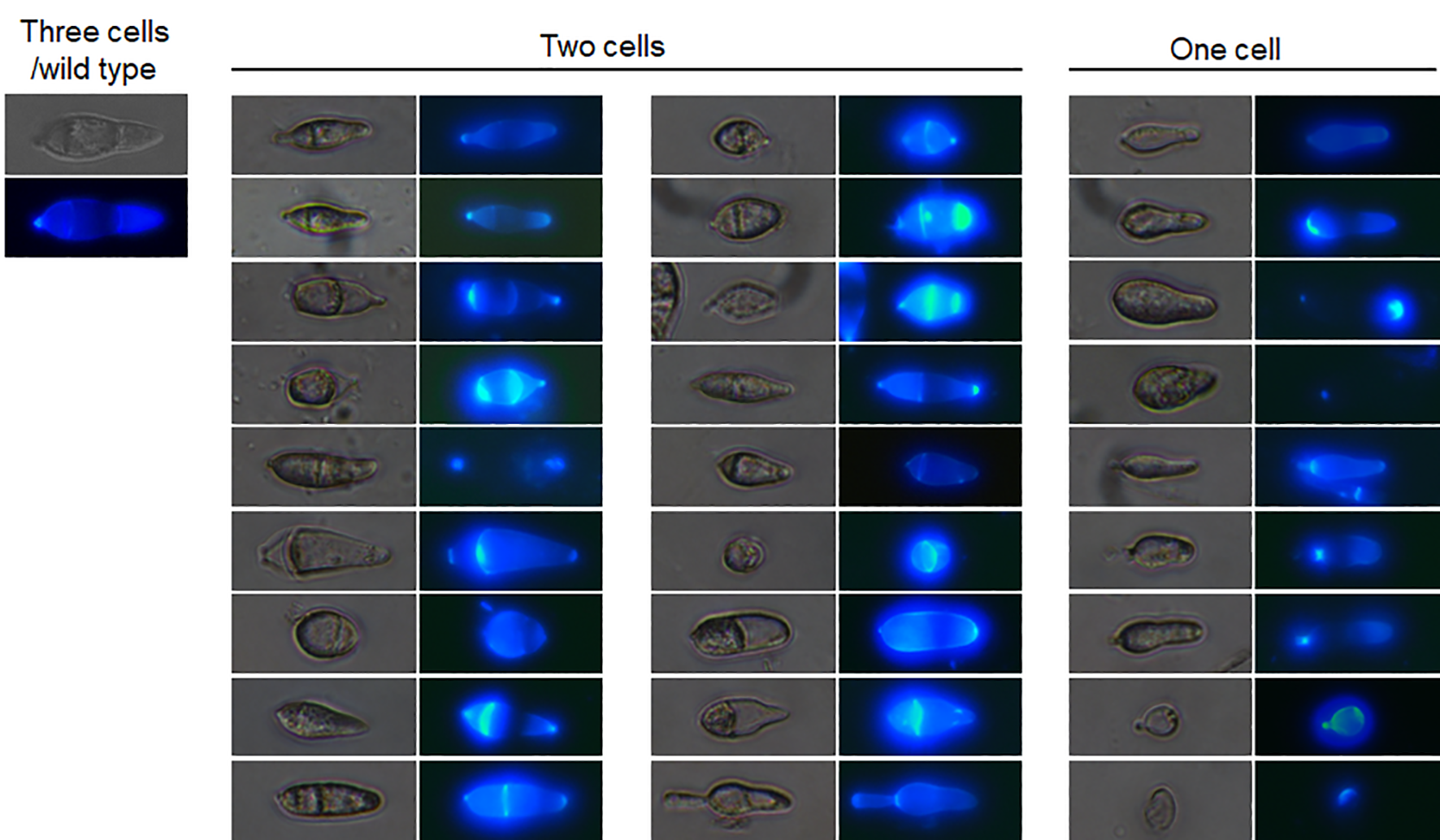


**Fig. S5 Conidial septa of the *RAM1* deletion mutants stained with CW.**

Supplement: Supplementary file 5 — Fig. S5 Conidial septa of the RAM1 deletion mutants stained with Calcofluor White (CFW). [file MPP-20-1264-s005.doc]
