# Supplementary material for: The farnesyltransferase β‐subunit RAM1 regulates localization of RAS proteins and appressorium‐mediated infection in Magnaporthe oryzae
Source: Mol Plant Pathol. 2019 Jun 27;20(9):1264–78. doi: 10.1111/mpp.12838 (PMC6715606; doi:10.1111/mpp.12838)
Supplement: Supplementary file 6 — Fig. S6 Invasive growth of invasive hyphae restored by diphenylene iodonium (DPI) treatment. Barley leaves were treated with or without DPI (0.5 mM) dissolved in DMSO. Invasive growth was observed at 30 hpi. Bars, 20 μm. [file MPP-20-1264-s006.doc]

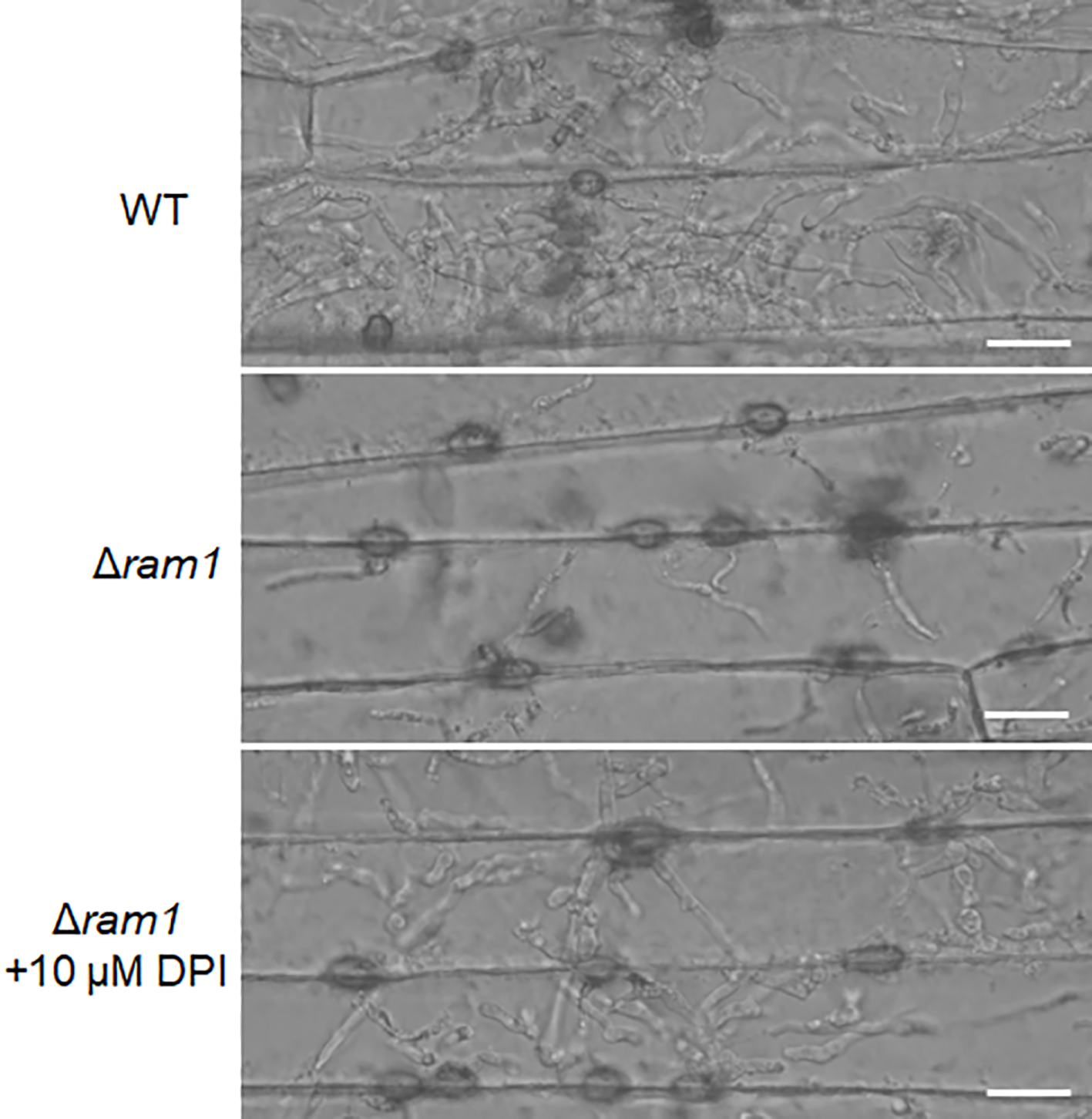


**Fig. S6. Invasive growth of IH restored by DPI treatment.** Barley leaves were treated with or without DPI (0.5 mM) dissolved in DMSO. Invasive growth was observed at 30 hpi. Bars, 20 μm.
