# Supplementary material for: The farnesyltransferase β‐subunit RAM1 regulates localization of RAS proteins and appressorium‐mediated infection in Magnaporthe oryzae
Source: Mol Plant Pathol. 2019 Jun 27;20(9):1264–78. doi: 10.1111/mpp.12838 (PMC6715606; doi:10.1111/mpp.12838)
Supplement: Supplementary file 7 — Fig. S7 Farnesylation site prediction in the C‐terminal of the RAS proteins in Magnaporthe oryzae. The farnesylation sites were predicted by GPS‐Lipid (http://lipid.biocuckoo.org/webserver.php) (Xie et al., 2016). The CaaX motifs are indicated in blue and the farnesylation sites are indicated in red. [file MPP-20-1264-s007.doc]

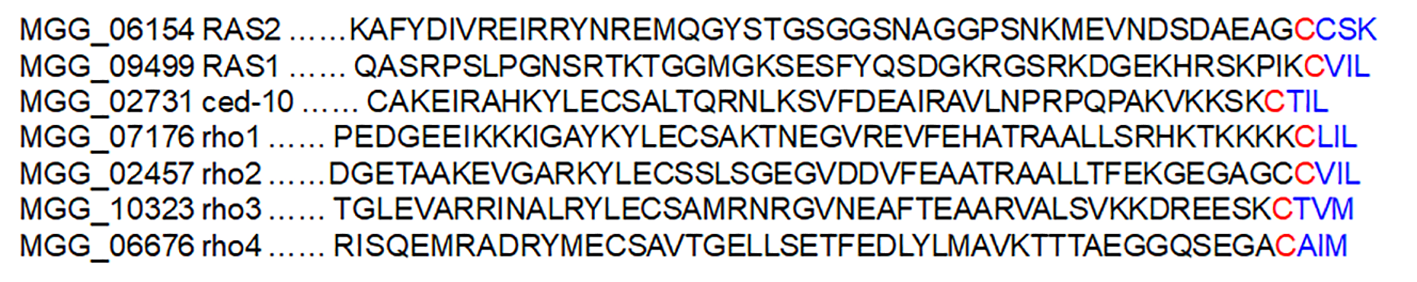


**Fig. S7 Farnesylation sites prediction in C-terminal of the RAS proteins in *M. oryzae*.** The farnesylation sites were predicted by GPS-Lipid (http://lipid.biocuckoo.org/webserver.php) (Xie et al., 2016). The CaaX motifs were indicated by blue, and the farnesylation sites were indicated by red.
