# Supplementary material for: The farnesyltransferase β‐subunit RAM1 regulates localization of RAS proteins and appressorium‐mediated infection in Magnaporthe oryzae
Source: Mol Plant Pathol. 2019 Jun 27;20(9):1264–78. doi: 10.1111/mpp.12838 (PMC6715606; doi:10.1111/mpp.12838)
Supplement: Supplementary file 9 — Fig. S9 Subcellular localization of RAS proteins in invasive hypha. WT/GFP:RAS1, the wild‐type strain expressing eGFP:RAS1; Δram1/GFP:RAS1, the Δram1 mutant expressing eGFP:RAS1; WT/GFP:RAS1C238S, the wild‐type strain expressing eGFP:RAS1C238S. WT/GFP:RAS2, the wild‐type strain expressing eGFP:RAS2; Δram1/GFP:RAS2, the Δram1 mutant expressing eGFP:RAS2; WT/GFP:RAS2C211S, the wild‐type strain expressing eGFP:RAS1C211S. Bars, 10 μm. [file MPP-20-1264-s009.doc]

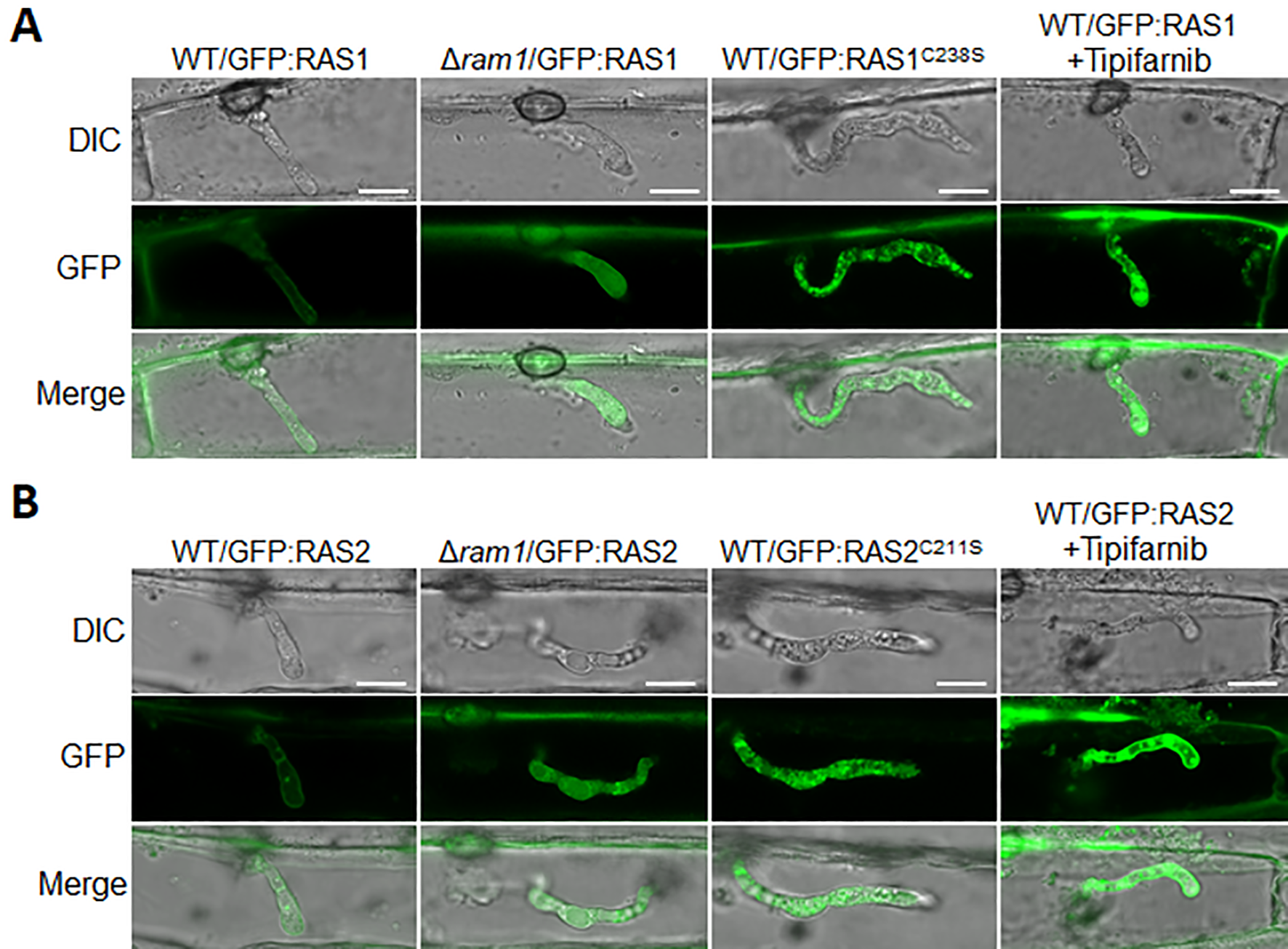


**Fig. S9. Subcellular localization of RAS proteins in invasive hypha.** WT/GFP:RAS1, the wild type strain expressing eGFP:RAS1; Δ*ram1*/GFP:RAS1, the Δ*ram1* mutant expressing eGFP:RAS1; WT/GFP:RAS1^C238S^, the wild type strain expressing eGFP:RAS1^C238S^. WT/GFP:RAS2, the wild type strain expressing eGFP:RAS2; Δ*ram1*/GFP:RAS2, the Δ*ram1* mutant expressing eGFP:RAS2; WT/GFP:RAS2^C211S^, the wild type strain expressing eGFP:RAS1^C211S^. Bars, 10 μm.
