# Supplementary material for: The farnesyltransferase β‐subunit RAM1 regulates localization of RAS proteins and appressorium‐mediated infection in Magnaporthe oryzae
Source: Mol Plant Pathol. 2019 Jun 27;20(9):1264–78. doi: 10.1111/mpp.12838 (PMC6715606; doi:10.1111/mpp.12838)
Supplement: Supplementary file 10 — Table S1 Fungal strains used in this study. [file MPP-20-1264-s010.docx]

**Table S1 Fungal strains used in this study.**

| **Strains** | **Genotypes** | **References** |
| --- | --- | --- |
| P131 | A wild-type isolate of *M. oryzae* | (Peng and Shishiyama, 1988) |
| KO4, KO6 | *RAM1* deletion mutants of P131, Δ*ram1*. | This study |
| cRAM1 | Complement strain of Δ*ram1*, Δ*ram1/RAM1* | This study |
| RAM1G | P131 transformed by *eGFP-RAM1* fusion construct, WT/GFP:RAM1. | This study |
| RAS1G | P131 transformed by *eGFP-RAS1* fusion construct, WT/GFP:RAS1. | This study |
| ram1/RAS1G | Δ*ram1* transformed by *eGFP-RAS1* fusion construct, Δ*ram1*/GFP:RAS1. | This study |
| RAS1G-M | Δ*ram1* transformed by *eGFP-RAS1*^C238S^ fusion construct, Δ*ram1*/GFP:RAS1^C238S^. | This study |
| RAS2G | P131 transformed by *eGFP-RAS2* fusion construct, WT/GFP:RAS2. | This study |
| ram1/RAS2G | Δ*ram1* transformed by *eGFP-RAS2* fusion construct, Δ*ram1*/GFP:RAS2. | This study |
| RAS2G-M | Δ*ram1* transformed by *eGFP-RAS2*^C211S^ fusion construct, Δ*ram1*/GFP:RAS2^C211S^. | This study |
| CoIP-RAS1 | P131 transformed by *eGFP-RAS1* and Ram1-3xFLAG constructs. | This study |
| CoIP-RAS2 | P131 transformed by *eGFP-RAS2* and Ram1-3xFLAG constructs. | This study |
