# Supplementary material for: The farnesyltransferase β‐subunit RAM1 regulates localization of RAS proteins and appressorium‐mediated infection in Magnaporthe oryzae
Source: Mol Plant Pathol. 2019 Jun 27;20(9):1264–78. doi: 10.1111/mpp.12838 (PMC6715606; doi:10.1111/mpp.12838)
Supplement: Supplementary file 11 — Table S2 Plasmids used in this study. [file MPP-20-1264-s011.doc]

**Table S2 Plasmids used in this study.**

| **Names** | **Descriptions** |
| --- | --- |
| pKN | Vector used to construct complementation vectors and other vectors; with the *NPTII* gene as a selective marker inserted into pKS^+^ (Yang et al., 2010). |
| pKN-*RAM1* | *RAM1* complementation vector; *RAM* gene containing 1.5 kb promoter and 0.5 kb terminator regions were amplified and inserted into pKN. |
| pKNRG | Vector used to construct vectors to constitutively express selected genes; with the fungal constitutive promoter RP27 (Yang et al., 2010). |
| pKNRG-*RAM1* | Vector for sub-cellular localization of RAM1 protein; coding region of *RAM1* was cloned into vector pKNRG. |
| pKNRG-*RAS1* | Vector for sub-cellular localization of RAS1protein; coding region of *RAS1* was cloned into vector pKNRG. |
| pKNRG-*RAS2* | Vector for sub-cellular localization of RAS2 protein; coding region of *RAS2* was cloned into vector pKNRG. |
| pKNRG-*RAS1^C238S^* | Vector for sub-cellular localization of RAS1 protein with mutation at 238 from C to S. |
| pKNRG-*RAS2^C211S^* | Vector for sub-cellular localization of RAS2 protein with mutation at 211 from C to S. |
| pKNFLAG | Vector used to construct vectors expressing 3xFLAG fusing protein promoted by constitutive promoter RP27. |
| pKNFLAG-*RAM1* | Vectors constitutively expressing *RAM1*-3xFLAG fusing protein. |
| pYES2 | Yeast expressing vector for inducible expression of recombinant proteins in *S. cerevisiae*. |
| pYES2-*RAM1* | Yeast complement vector for inducible expression of Ram1 recombinant proteins. |
