# Supplementary material for: The farnesyltransferase β‐subunit RAM1 regulates localization of RAS proteins and appressorium‐mediated infection in Magnaporthe oryzae
Source: Mol Plant Pathol. 2019 Jun 27;20(9):1264–78. doi: 10.1111/mpp.12838 (PMC6715606; doi:10.1111/mpp.12838)
Supplement: Supplementary file 12 — Table S3 Primers used in this study. [file MPP-20-1264-s012.doc]

**Table S3 Primers that used in this study.**

| **Primers** | **Sequences (5'–3')** |
| --- | --- |
| HPT-LCK | GACAGACGTCGCGGTGAGTT |
| HPT-RCK | TCTGGACCGATGGCTGTGTAG |
| HPT-F1 | CTCCGACCTGATGCAGCTCT |
| HPT-R1 | CTCGCTCCAGTCAATGACC |
| HYG-LB | ACCTCCACTAGCTCCAGCCAAG |
| HYG-RB | GAATAGAGTAGATGCCGACCGGG |
| RAM1LBCK | GGCTGGCCAAGGATCTGTAG |
| RAM1LB-F | GGTGAGCACTTCTGTAGTTGG |
| RAM1LB-R | ACCTCCACTAGCTCCAGCCAAGGACAACGGTGCCCATTGC |
| RAM1RB-F | GAATAGAGTAGATGCCGACCGGGAGTGAGGTTATGCCGAGA |
| RAM1RB-R | TTGATTCACTCTCCCACTCC |
| RAM1RBCK | ACTGGGATTCGCTAGCCTCG |
| RAM1HPT-F | ATGGGCACCGTTGTCCTTGGCTGGAGCTAGTGG |
| RAM1GFP-F | TTTGAATTCATGAGACACCACACAAAG |
| RAM1GFP-F | TTTGAATTCATGGATTCTTCCTCCTC |
| RAM1GFP-R | TTCGGATCCTTAGAATCCATCTCTTGA |
| RAS1-GFPF | CAGATCGAATTCCTGCCCGGGGTCATGACTGGAAGGTTGCA |
| RAS1-GFPR | ACGTTAAGTGGATCCCCCGGGGAGGCTCACAATATAACACACTTG |
| RAS1m-GFPF | CAGATCGAATTCCTGCCCGGGGTCATGACTGGAAGGTTGCA |
| RAS1m-GFPR | ACGTTAAGTGGATCCCCCGGGGAGGCTCACAATATAACACACTTG |
| RAS2-GFPF | TTCCTGCAGCCCGGGGGATCCATGGCTCAGTCAAAGGTTCG |
| RAS2-GFPR | TCAGTAACGTTAAGTGGATCCTTACATCAAGACACACTTGGAGC |
| RAS2m-GFPF | TTCCTGCAGCCCGGGGGATCCATGGCTCAGTCAAAGGTTCG |
| RAS2m-GFPR | TCAGTAACGTTAAGTGGATCCTTACATCAAGACACACTTGGAGC |
| RAM1qF | GTTGGGGATGACGACATG |
| RAM1qR | AGAATCCATCTCTTGAAG |
| RAM1FLAG-F | TTTGAATTCATGGATTCTTCCTCCTC |
| RAM1FLAG-R | CGACCCGGGTTAGAATCCATCTCTTGA |
| RAM1PGPBKT7-F | TTTGAATTCATGGATTCTTCCTCCTC |
| RAM1PGPBKT7-R | ACGGATCCTTAGAATCCATCTCTTGA |
| RAS1PGADT7-F | CATCGATACATGACTGGAAGGTTGCA |
| RAS1PGADT7-R | AGCTCGAGCTCACAATATAACACACTTG |
| RAS2PGADT7-F | GCAGAATTCATGGCTCAGTCAAAGGTTCG |
| RAS2PGADT7-R | GTATCGATGTTACATCAAGACACACTTGGAGC |
| RAM1pYES-F | ATGGATCCAATGGATTCTTCCTCCTC |
| RAM1pYES-R | TCAGAATTCTTAGAATCCATCTCTTGA |
